# TiO_2_ and CaCO_3_ Microparticles Produced in Aqueous Extracts from *Satureja montana:* Synthesis, Characterization, and Preliminary Antimicrobial Test

**DOI:** 10.3390/molecules30204138

**Published:** 2025-10-20

**Authors:** Federica Valentini, Irene Angela Colasanti, Camilla Zaratti, Dumitrita Filimon, Andrea Macchia, Anna Neri, Michela Relucenti, Massimo Reverberi, Ivo Allegrini, Ettore Guerriero, Marina Cerasa, Marta De Luca, Francesca Santangeli, Roberto Braglia, Francesco Scuderi, Lorenza Rugnini, Roberta Ranaldi, Roberto De Meis, Antonella Canini

**Affiliations:** 1 Sciences and Chemical Technologies Department, Tor Vergata University, Via della Ricerca Scientifica 1, 00133 Rome, Italy; ireneangela.colasanti@students.uniroma2.eu (I.A.C.); camilla.zaratti@students.uniroma2.eu (C.Z.); filimon.1768827@studenti.uniroma1.it (D.F.); 2 YOCOCU APS, Via Torquato Tasso 108, 00185 Rome, Italy; 3 Department of Biology, Ecology and Earth Sciences (DiBEST), University of Calabria, Via Pietro Bucci, 87036 Arcavacata, Italy; aps@yococu.com; 4 Department of Biomedicine and Prevention, Tor Vergata University, Viale Montpellier 1, 00133 Rome, Italy; anna.neri@uniroma2.it; 5 Department of Anatomical, Histological, Forensic and Orthopaedic Sciences, Sapienza University of Rome, Via Alfonso Borelli 50, 00161 Rome, Italy; michela.relucenti@uniroma1.it; 6 Department of Environmental Biology, Sapienza University of Rome, Piazzale Aldo Moro 5, 00185 Rome, Italy; massimo.reverberi@uniroma1.it; 7 Envint Srl, Via Paradiso 65a, Montopoli di Sabina, 02034 Rieti, Italy; ivo.allegrini@tiscali.it; 8 Institute of Atmospheric Pollution Research (IIA), National Research Council (CNR), Strada Provinciale 35d, 9, 00010 Montelibretti, Italy; ettore.guerriero@cnr.it (E.G.); marina.cerasa@cnr.it (M.C.); 9 Department of Physics, Sapienza University of Rome, Piazzale Aldo Moro 2, 00185 Rome, Italy; marta.deluca@uniroma1.it (M.D.L.); francesca.santangeli@uniroma1.it (F.S.); 10 Department of Biology, Tor Vergata University of Rome, Via della Ricerca Scientifica 1, 00133 Rome, Italy; roberto.braglia@uniroma2.it (R.B.); francesco.scuderi@uniroma2.it (F.S.); lorenza.rugnini@uniroma2.it (L.R.); ranaldiroberta@gmail.com (R.R.); roberto.demeis@gmail.com (R.D.M.); canini@uniroma2.it (A.C.)

**Keywords:** *Satureja montana*, plant extract-functionalized microparticles, GC-MS extract analysis, oxygenated terpenoids as activator/phase controllers, terpinene, thymoquinone, carvacrol, thymol, l-linalool, preliminary antimicrobial screening

## Abstract

The possibility of modifying the surface chemistry of materials and synthetizing inorganic particles in natural aqueous extracts of plants (avoiding calcination), opens the doors to undoubtedly interesting scenarios for innovative functionalization strategies that are increasingly eco-sustainable and rich in interesting chemical–physical and biochemical properties. Among the aerial plants, *Satureja montana* exhibits interesting antibacterial, antifungal, antimicrobial, and antioxidant activities due to the rich volatile and non-volatile compounds (characterized by gas chromatography–mass spectrometry), contained in the aqueous extracts. For the first time, the latter was applied for the green synthesis of TiO_2_ and CaCO_3_ particles, characterized by X-ray diffraction, Raman, infrared spectroscopies, and scanning electron microscopy, coupled with microanalysis. Screening through antimicrobial assays under indoor passive sedimentation conditions showed opposite trends for two kinds of particles. TiO_2_ anatase spherical particles (400 < φ < 600 nm) increase microbial growth, proportionally to increasing particle concentration. Instead, *S. montana*-functionalized CaCO_3_ prismatic microparticles (1 µm × 1 µm × 1 µm) exhibit strong and dose-dependent antimicrobial activities, achieving near-complete inhibition at 50 mg/mL.

## 1. Introduction

**Scheme 1:**
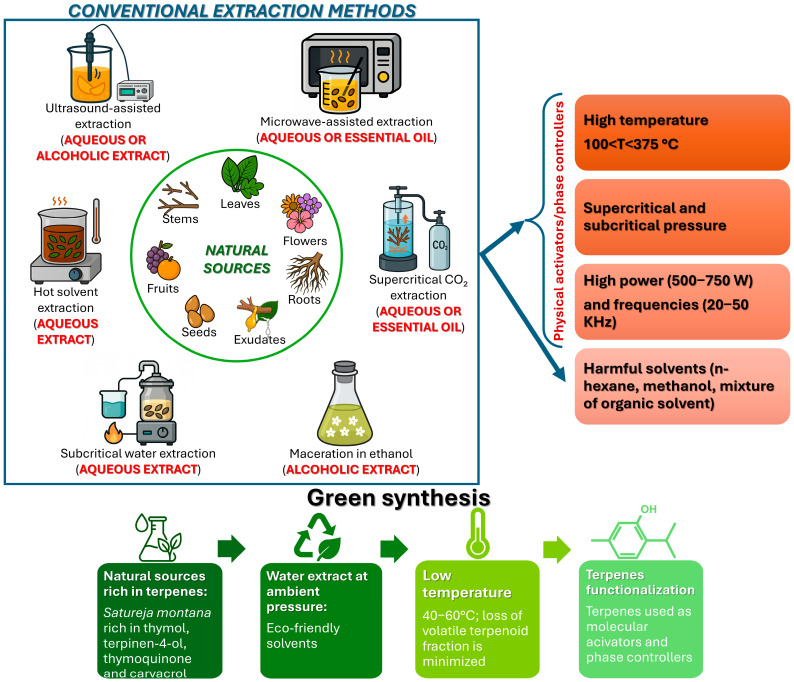
Comparison between extraction methods using physical activators and the method proposed in this study, where the activators are chemical molecules.

The possibility of synthesizing and especially functionalizing micro- and nanomaterials in eco-sustainable work environments certainly opens the doors to new and interesting possibilities in obtaining smart nanomaterials with extraordinary chemical–physical [1], biochemical [2], and medical properties [3] of great relevance. For a long time, surface chemistry has been exclusively concerned with predominantly chemical functionalization reactions. The great variety of natural substances, such as essential oils [4] and natural extracts [5] from medicinal plants [6], allows (in large-scale mass production) increasingly eco-friendly micro- and nanomaterials to be obtained; these are characterized by excellent properties/features of clinical–medical [7], environmental [8], cultural heritage restoration/conservation [9], and food packaging interest [10]. Recently, several interesting review papers provided innovative advancements in green synthesis strategies to produce eco-friendly materials (see Scheme 1), focusing on raw materials, their reaction mechanism and surface chemistry, and the challenges involved in different application fields [11,12,13].

The strategies based on the green synthetic approach offer several advantages over the traditional mechanism of production of new materials (as reported in Scheme 1).

In Scheme 1, it is clearly evident that from the leaves, roots, flowers, and fruits of many natural medicinal plants, it is possible to obtain natural plant extracts (NPEs), concentrated essential oils (EOs), resins, and exudates capable of acting as “templates” in the nucleation and growth of inorganic functionalized particles [14]. Still describing the information that can be deduced from Scheme 1, there are different methods, which are very well-known and reported in recent literature [15,16], to obtain these extracts from several medicinal/officinal plants. In all the plant extraction methods illustrated in Scheme 1, the working temperature is greater than or equal to 100 °C. In particular, in the subcritical water extraction treatment, the temperature can reach values up to 375 °C, and pressure high enough to keep water in the liquid state can also be applied [17]. In other methodologies, such as the one based on supercritical CO_2_ extraction, the working conditions are the supercritical CO_2_ ones (T = 31.1 °C and P = 73.8 bar) [18]. Other methods that obtain oils, resins, etc., from natural plants through hot extraction (carried out at T > 100 °C) employ organic solvents or mixtures of these (such as hexane/ethanol) [19]. The presence of organic solvents is however poorly compatible for applications in the most diverse analytical fields, ranging from food, medicine, agriculture, and biotechnology [20,21]. Methods based on microwave treatment couple high temperatures with working powers of around 500 Watts [22]. And finally, the methods that apply ultrasonication also work at T > 100 °C, applying powers in the range of 500–750 Watts and high ultrasound frequencies [23]. In all these extraction methods presented in Scheme 1, the experimental conditions described are those that compromise the presence of the volatile fraction of terpenes, and the only physical parameter that actually controls the shape, size, and defectivity of the resulting particles is temperature/T. In this way, control of the induced defects, and therefore of the functionalization of the particles synthesized in extracts and/or EO of natural plants, is also lost.

Thus, in accordance with what was stated above, the novelty of this work is to perform an aqueous extraction of natural substances from *Satureja montana* (SM), and a subsequent synthesis of TiO_2_/anatase and CaCO_3_/calcite particles at T < 100 °C (especially in the range from 40 to 60 °C). The procedure favors the presence of terpenes anchored to the particles growing in these extracts (used here as working media), minimizing the loss of the volatile fraction. This latter becomes the true molecular chemical activator/phase controller in the growth of the inorganic particles, providing them with the antimicrobial activities and other features. A very high concentration of oxygenated terpenoids, such as carvacrol, thymol, etc., is contained in SM [24], and for this reason our choice fell on this medicinal plant. These terpenes are anchored on the surface of TiO_2_ and CaCO_3_ particles, by regulating its shape, size, and the presence of chemically active defects/edges. The real challenge was to produce TiO_2_/anatase without calcining, functionalizing it, for the first time, with terpenes extracted from SM [25] and to study its antimicrobial properties.

The other completely innovative aspect of this study is the production of CaCO_3_/calcite particles with antimicrobial activity, knowing already that calcite does not intrinsically possess antibacterial/antimicrobial properties. In fact, it is known that only when calcium carbonate is in the form of nanoparticles (using high-pressure hydrothermal treatment) or when it is incorporated into composites can antimicrobial activities be demonstrated, including inhibiting the growth of bacteria like *Escherichia coli* and *Staphylococcus aureus* [26]. This study allows the green fabrication of CaCO_3_/calcite particles in aqueous SM extract, conferring antimicrobial activity by anchoring oxygenated terpenes on the particle surface, avoiding treatments that require extreme temperature and pressure conditions. This would make calcite an excellent fertilizer carrier in agriculture [27,28] and also a very powerful tool for the delivery of active ingredients in many fields of application [29].

Finally, to understand and read the text clearly, Table S1 (in Supplementary Information) reports the nomenclature and abbreviations of all the chemical compounds considered in the manuscript.

## 2. Results and Discussion

This chapter is organized as follows: first, the results related to the morphological and structural characterization of the new functionalized microparticles are reported and described. Then, the chemical composition of the natural SM extracts is discussed, by means of GC-MS analysis; and finally, the results related to the study of the antibacterial, antimicrobial, antioxidant, and antibiofilm properties, found in appropriate bacterial strains selected and treated at different concentrations of new functionalized particles, are widely reported and discussed.

### 2.1. Functionalized Microparticles and Their Characterization Study

#### 2.1.1. TiO_2_ MPs/SM

**Figure 1:**
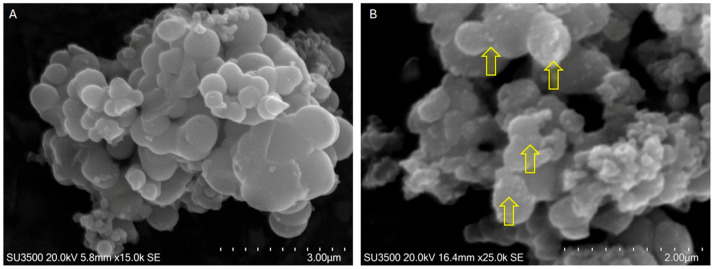
SEM of TiO_2_ microspheres. (**A**) A microsphere cluster is presented, spherical structures are visible, with variable sizes ranging from 160 nm up to 1.3 µm, magnification 10 K. (**B**) A higher magnification of a microsphere cluster shows the amorphous organic material on the surface of microparticles (arrows), magnification 25 K.

The use of certain plant extracts and optimized synthesis conditions has been shown in previous studies to promote anisotropic growth of TiO_2_ nanostructures, leading to morphologies such as nanorods [30] and nanocubes or ellipsoidal forms [31]. Therefore, a non-spherical structure might have been expected under specific conditions. However, in our case, the TiO_2_ MPs/SM synthesized using the selected extract and protocol predominantly exhibit a spherical morphology, with diameters ranging from 160 nm to 1.3 μm and the majority falling between 400 and 600 nm (Figure 1A). This outcome suggests that, under the conditions employed, the extract did not exert a strong shape-directing effect or, alternatively, that the reaction parameters favored isotropic growth. This observation aligns with other reports in which TiO_2_ particles synthesized via green methods also adopted spherical morphologies when shape-directing forces were not dominant. The surface of the micro/nanospheres appears smooth and the observations at high magnification (Figure 1B) suggest that the spherical elements have variable sizes because they arise from the nucleation of TIO_2_ around a core of amorphous organic substance and then they gradually growth in size by TIO_2_ apposition. The amorphous aggregates of organic matter are indicated by the arrows in Figure 1B.

**Figure 2:**
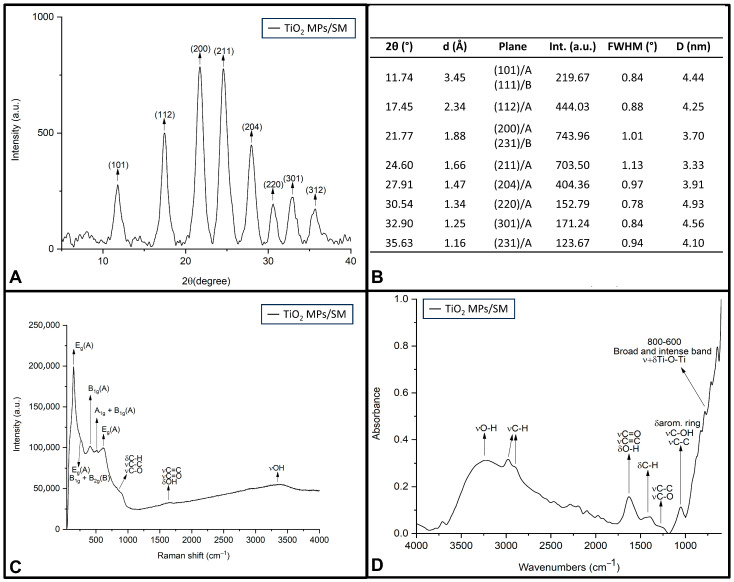
TiO_2_ MPs/SM. XRD spectrum (**A**) and quantitative XRD table (**B**), Raman (**C**), and FTIR (**D**).

The XRD analysis is reported in Figure 2A, with all the typical peak assignments, attributable to anatase (about 90% of the sample) (ICDD Card No. 01-075-2552), and all the quantitative measurements for TiO_2_ crystals are summarized in Figure 2B.

Raman spectroscopy provided further confirmation of the TiO_2_ crystalline phases (Figure 2C and Table 1). The spectrum revealed the presence of characteristic bands of the anatase polymorph, confirming the results obtained by XRD analysis. The Raman spectrum also exhibited broad fluorescence-related features at ~874, ~1627, and ~3352 cm^−1^, which are likely associated with the vibrational modes of the organic functional groups/phase controllers used to functionalize/draw the particles (see Figure S1 in Supplementary Information). FTIR-ATR analysis (Figure 2D and Table 1) confirms the anchoring of oxygenated functional groups (identified by GC-MS analysis, reported later in the text), mainly of an alcoholic and carbonyl nature (such as aldehydes and ketones).

Further details on XRD, Raman, and FTIR discussions are reported in the Supplementary Information, respectively, for TiO_2_ MPs/SM. The Supplementary Information also contains all the detailed morphological characterization of the pristine (nano-sized) TiO_2_ control sample.

#### 2.1.2. CaCO_3_ MPs/SM

**Figure 3:**
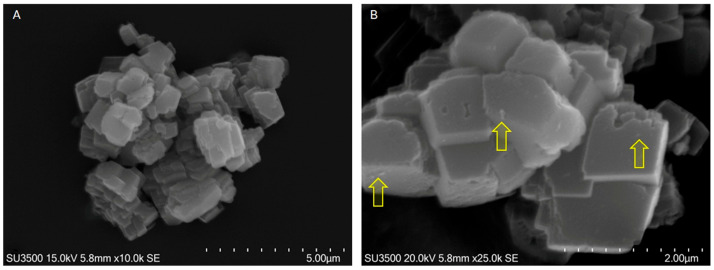
SEM of CaCO_3_ crystal. (**A**) A crystal cluster is presented, prismatic structures with sharp edges are visible, one on the top of each other, magnification 10 K. (**B**) A higher magnification of a crystal cluster shows the amorphous organic material on the surface of prismatic crystals (arrows), and evidence that they are formed by a layered structure of thin plates, magnification 25 K.

The CaCO_3_ particles in the examined alcohol suspension appear as crystals with a prismatic shape, sharp edges, and variable size (Figure 3A), with an average of 1 µm × 1 µm × 1 µm. Amorphous aggregates of organic matter (indicated by the arrows in Figure 3B) are adherent onto the smooth surface of the crystals. The image B (high magnification) shows that the prismatic crystals’ structure is the result of a stratification of thin and smooth plates (like cheddar cheese layered tiles).

**Figure 4:**
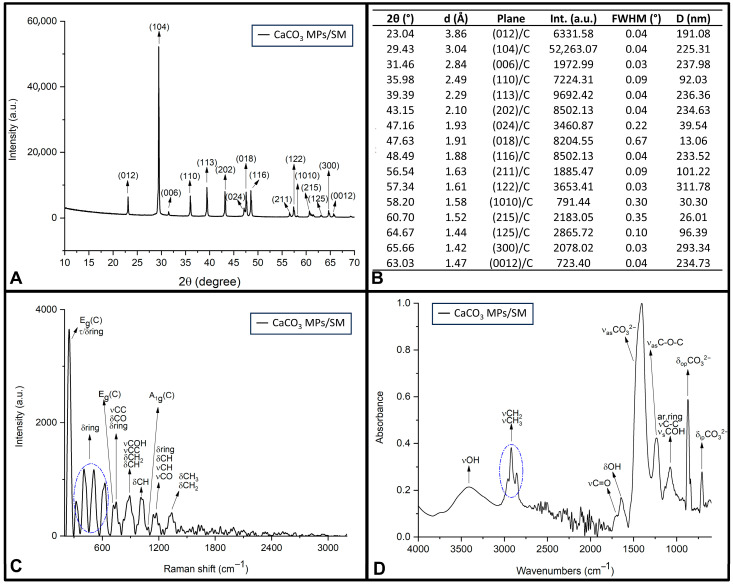
CaCO_3_ MPs/SM. XRD spectrum (**A**) and quantitative XRD table (**B**), Raman (**C**), and FTIR (**D**).

The diffractogram obtained for CaCO_3_ MPs/SM reveals calcite as the predominant crystalline phase (Figure 4A), and all the quantitative measurements, traceable from the diffractogram of Figure 4A, are accurately reported in Figure 4B.

Complementary insights were provided by Raman spectroscopy, which revealed vibrational features attributable to both the calcite phase and organic phytocompounds introduced by the SM extract during synthesis (Figure 4C and Table 2). The FTIR spectrum further confirms the dual nature of the material, revealing contributions from both the inorganic carbonate compound and the organic functionalities (Figure 4D and Table 2). There is a significant difference with the functional groups anchored on the CaCO_3_ MPs/SM, which are predominantly of an acidic carboxylic nature (C(=O)O-H), compared to those identified for TiO_2_ MPs/SM, which are of a carbonyl nature (aldehydes and ketones). In particular, the attributions marked in bold in Table 2 show the typical FTIR signals of unsaturated cyclic carbonates, where due to the conjugation effect between the carbonate and the unsaturated rings (and/or allene molecules) of the terpenoids, the absorbance signals appear at lower vibration frequencies (because they are stabilized by the effect of double bonds), in agreement with the literature [54].

Further details on the XRD, Raman, and FTIR attributions are given in the Supplementary Information.

### 2.2. Chemical Composition of NPEs from SM by GC-MS

GC-MS analyses were performed using both targeted and untargeted approaches to comprehensively characterize the volatile fraction of the SM aqueous extract. The integration of both methods allowed not only the identification of a defined set of known volatile constituents but also the detection of additional, previously unconsidered compounds, potentially involved in the green synthesis and surface functionalization of microparticles.

#### 2.2.1. Targeted Analysis

A targeted GC-MS analysis was performed on the aqueous extract of SM immediately after preparation, with the aim of characterizing its volatile composition and identifying compounds potentially involved in the surface functionalization of microparticles. The analysis was carried out using a calibrated terpene standard mixture, allowing the semi-quantitative determination of 24 selected volatile organic compounds (see Table 3).

The extract was dominated by oxygenated monoterpenes, with thymol being by far the most abundant constituent (63,906 ng/mL), followed by terpinen-4-ol (10,305 ng/mL) and thymoquinone (8271 ng/mL). Other noteworthy compounds included γ-terpinene (882 ng/mL), carvacrol (5669 ng/mL), α-terpinene (3814 ng/mL), and sabinene (1054 ng/mL). The presence of α-thujone (282 ng/mL), α-phellandrene (264 ng/mL), and α-terpinolene (284 ng/mL) further confirmed the richness of the terpene fraction. Trace amounts of carvacrol methyl ether (23 ng/mL), caryophyllene oxide (135 ng/mL), L-linalool (1425 ng/mL), and para-cymene (157 ng/mL) were also identified.

The volatile profile of the extract included monoterpene hydrocarbons (e.g., sabinene, α-thujone, α-terpinene), oxygenated derivatives (e.g., thymol, terpinen-4-ol, caryophyllene oxide), and aromatic compounds (e.g., *p*-cymene, thymoquinone), reflecting a chemically diverse composition.

Notably, thymol and terpinen-4-ol showed the highest peak areas, followed by thymoquinone, carvacrol, and α-terpinene, while compounds like caryophyllene oxide and l-linalool were present at lower levels. This distribution confirms the predominance of oxygenated monoterpenes among the most abundant constituents.

The analysis was conducted on a single, well-characterized extract, providing a representative chemical fingerprint under the tested conditions. Although the lack of replicates prevents statistical evaluation, the dataset offers a consistent reference for subsequent applications using the same preparation.

#### 2.2.2. Untargeted Analysis

In addition to the targeted screening, a non-targeted GC-HRMS approach was employed to comprehensively explore the chemical complexity of the SM aqueous extract and to identify additional constituents potentially involved in the green synthesis and surface functionalization of microparticles. This strategy aimed to complement the targeted dataset by detecting molecules not included in the calibration mixture but potentially relevant in mechanistic or functional terms.

**Figure 5:**
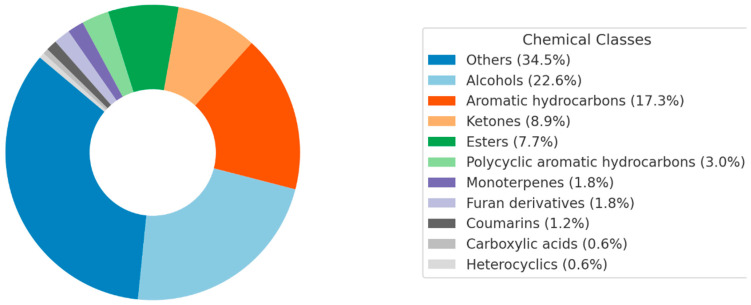
Chemical classification of high-confidence compounds identified in the untargeted GC-HRMS analysis of SM aqueous extract. The chart is based on the compound list reported in Supplementary Table S1, where molecules were grouped into chemical families according to their molecular structures and functional groups.

Following peak deconvolution, a total of 643 individual features were detected. After procedural blank subtraction and spectral quality filtering (Total Score > 90), 169 compounds were identified with high confidence. These were subsequently categorized into major chemical families based on their molecular structures and functional groups. As shown in Figure 5, alcohols (22.6%), aromatic hydrocarbons (17.3%), and ketones (8.9%) were among the most represented classes. Esters, polycyclic aromatic hydrocarbons, monoterpenes, and furan derivatives were also identified, while a substantial fraction (34.5%) fell under the category “Others,” encompassing structurally ambiguous, multifunctional, or less-characterized phytochemicals. These results provide a broad overview of the chemical landscape of the extract and highlight its richness in oxygenated and aromatic species, which are likely to influence its reactivity and interaction with inorganic surfaces.

The full dataset, including retention times, average peak areas, molecular formulas, exact and theoretical masses, and NIST match scores, is reported in Supplementary Table S1. In many cases, compounds were detected at multiple retention times but shared the same molecular formula and theoretical mass. These signals, retained in the final table, likely represent structural or conformational isomers and are discussed collectively based on their elemental identity.

**Figure 6:**
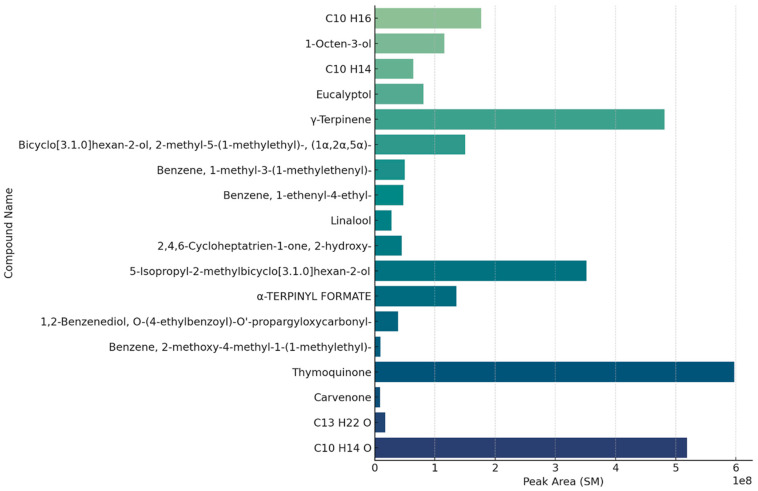
Histogram plot of the 22 most abundant compounds identified in the SM extract based on peak area, ordered by retention time. Thymol and endo-borneol were excluded from the graph due to their disproportionately high peak areas, which would distort the scale. Since it is a single sample, there is no standard deviation. The bars indicate the relative abundance of the compounds in the sample because they are chromatographic peak areas, not quantities.

To focus the discussion on the most relevant compounds, the 22 molecules with the highest peak areas—each with a Total Score > 95 and a Peak Rating > 7.5—were selected and are visualized in Figure 6. For clarity, thymol and endo-borneol, although confirmed as major constituents, were excluded from the graph due to their disproportionately high abundance, which would distort the scale. Their identity is confirmed and discussed elsewhere in the text.

The selected compounds span a broad retention time range and include molecules with well-defined structure as well as entries reported solely by molecular formula (e.g., C_10_H_16_, C_10_H_14_, C_13_H_22_O). These formula-based entries indicate the detection of multiple peaks with identical mass and elemental composition but distinct chromatographic behaviors, suggesting the presence of unresolved isomeric species. Although further structural elucidation is needed, such components are retained for their potential contribution to the extract’s reactivity and its role in downstream applications.

In the following paragraphs, the results regarding the application of TiO_2_ MPs/SM and CaCO_3_ MPs/SM in bacterial cultures are described, and the order of presentation will respect that followed for all other results previously shown.

### 2.3. Antimicrobial Screening

**Figure 7:**
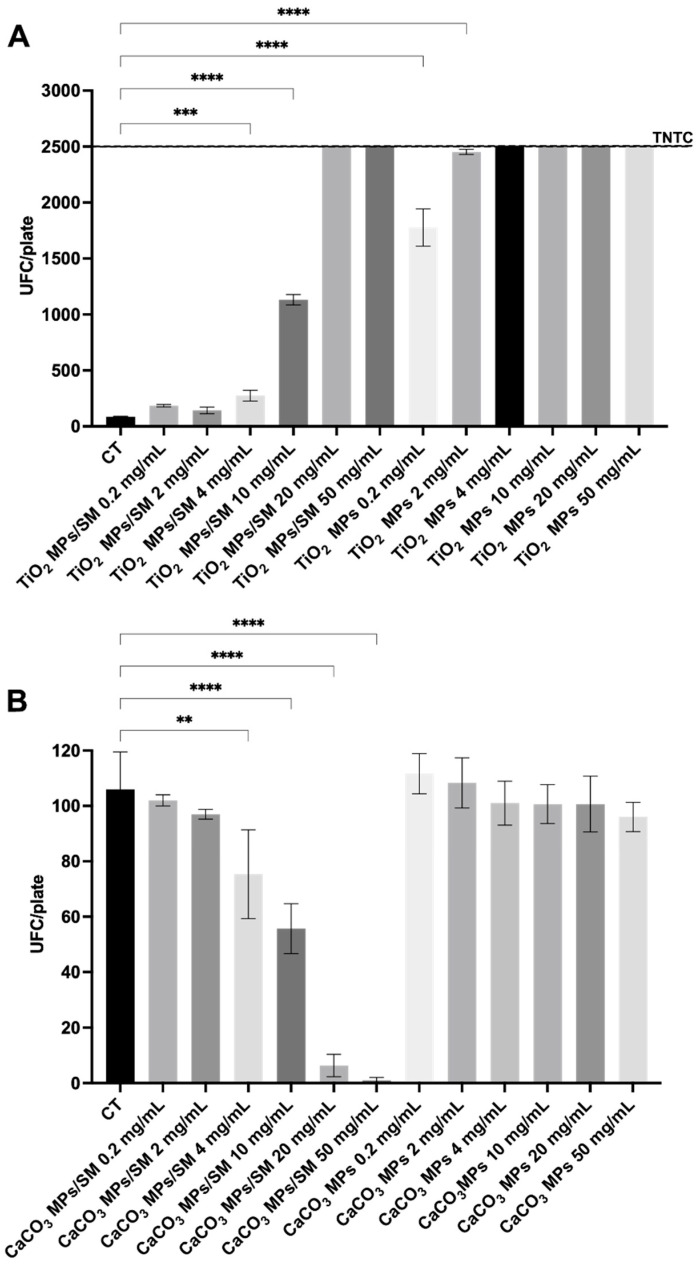
Mean ± SD (*n* = 6) of CFU/plate following passive sedimentation method under different TiO_2_ (**A**) and CaCO_3_ MPs (**B**) treatments. TNTC: too numerous to count (CFU > 2500); CT: untreated control. Statistical significance relative to the untreated control is indicated by asterisks: ** *p* < 0.005; *** *p* < 0.001; **** *p* < 0.0001.

In the present study, the antimicrobial activity of TiO_2_ and CaCO_3_ MPs was evaluated within an indoor environmental setting. The passive sedimentation method was employed, as this approach is widely regarded as one of the most cost-effective, standardized, and reproducible methods for such investigation [59]. The antimicrobial activity of TiO_2_ microparticles (MPs) and TiO_2_ MPs functionalized with SM leaf extract (TiO_2_ MPs/SM) was evaluated in comparison with the untreated control (CT) (Figure 7A). In the control condition, passive air sampling yielded 66.33 ± 5.51 CFU/plate, representing the baseline microbial load in the indoor environment. When TiO_2_ MPs/SM were applied, low to moderate concentrations (0.2–4 mg/mL) produced a statistically non-significant increase in CFUs compared to the control, indicating a limited impact on microbial deposition. However, at 10 mg/mL a statistically significant increase was observed, 1132.00 ± 46.13 (*p* < 0.0001), and at the highest concentrations (20–50 mg/mL), the number of CFUs was found to be too numerous to count (TNTC), suggesting an unexpected promotion of microbial accumulation rather than inhibition. A similar but more pronounced trend was observed for TiO_2_ MPs, where even the lowest dose (0.2 mg/mL) caused a dramatic increase in microbial growth, 1776.00 ± 166.40 CFU/plate (*p* < 0.0001), further escalating at 2 mg/mL (2785.00 ± 190.31 CFU/plate), with higher concentrations resulting in TNTC. Compared to the CT, both formulations failed to reduce microbial contamination. Contrary to what was observed with TiO_2_ MPs data regarding the efficiency of antibacterial activity, promising results were observed with CaCO_3_ MPs data, as shown in Figure 7B. Compared to the CT (106.00 ± 13.52 CFU/plate), the application of CaCO_3_ MPs and CaCO_3_ MPs/SM induced a dose-dependent modulation of microbial growth.

For CaCO_3_ MPs/SM, low concentrations (0.2–2 mg/mL) did not significantly differ from the control, maintaining CFU counts close to CT, respectively, of 102.00 ± 2.00 and 97 ± 1.73 CFU/plate. However, starting from 4 mg/mL, a statistically significant reduction was observed with a value of 75.32 ± 16.04 CFU/plate (*p* < 0.01), which became even more pronounced at 10 mg/mL with the total number of CFUs per plate equal to 55.67 ± 9.01 CFU/plate (*p* < 0.0001). At 20 mg/mL and 50 mg/mL, microbial growth was almost completely suppressed, showing values equal, respectively, to 6.33 ± 4.04 and 1 ± 1 CFU/plate (*p* < 0.0001), indicating a strong antimicrobial effect at high doses. In contrast, the application of CaCO_3_ MPs did not produce statistically significant reductions compared to CT at any tested concentration, with CFU counts ranging from 111.70 ± 7.23 at 0.2 mg/mL to 96.00 ± 5.29 at 50 mg/mL, thus remaining within the same range registered for CT.

The results are also summarized in Table 4.

The data presented in the table clearly demonstrate contrasting antimicrobial behaviors between TiO_2_- and CaCO_3_-based microparticles (MPs), particularly when functionalized with SM extract. TiO_2_ MPs, both in their pristine (as-prepared) and SM-functionalized forms, not only failed to reduce microbial contamination but exhibited a dose-dependent increase in colony-forming units (CFUs), with values exceeding the countable limit (>2500 CFU/plate) at concentrations ≥ 4 mg/mL. This suggests a possible aggregation or nutrient-like effect that supports microbial proliferation and cell growth. Instead, CaCO_3_ MPs/SM showed a clear dose-dependent reduction in microbial load, with statistically significant decreases in CFUs starting from 4 mg/mL and nearly complete inhibition at higher doses (20–50 mg/mL). It is worth noting that several studies have demonstrated the antibacterial properties of SM extracts and essential oils against a range of bacterial and fungal pathogens [60,61,62]. Nonetheless, a direct comparison with MPs is not feasible, since once the extract is bound to the particles its actual concentration and composition cannot be precisely defined. This work therefore focused on the antimicrobial response at the material level, where MPs provide the additional advantage of reducing the volatility of active compounds, extending their persistence in the environment, and potentially enhancing their efficacy while lowering the required amount of extract.

All this finds a reasonable and valid explanation in many factors (undoubtedly regulated by the molecular activators/phase controllers), such as the presence of the organic functional groups, chemically bound to the surface of the particles (considered as ROS precursors), which thus become more reactive towards various bacterial strains.

In particular, the photocatalytic activity of TiO_2_ microparticles is mainly determined by their crystalline phases (anatase has the highest photocatalytic activity), grain size, specific surface area, pore structure, crystallinity, defects, and edges [63]. The spherical microparticles characterized in this study have dimensions greater than 100 nm (see Figure 1); they appear completely smooth and without edges, and for this reason they are inactive in antimicrobial tests. The hydrodynamic diameter was quantitatively evaluated using DLS (see Table 5) for spherical TiO_2_ and was found to be larger than the diameter of the unsolvated solid particles. Furthermore, TiO_2_ MPs/SM do not exhibit significant roughness, as shown by the BET data (see Table 6) [64,65].

Similarly to what was observed for TiO_2_ MPs/SM, pristine TiO_2_ microparticles (MPs with 200 nm as diameter, see Table 5 and Table 6 and Supplementary Information for pristine particles characterization) with the same phase composition (anatase and smaller % of brookite) also do not show any antimicrobial activity at all. Then, the authors also tried to synthesize a mixed phase/heterophase brookite/anatase (with a higher % of brookite and lower % of anatase) [66], but even in this case, no significant antimicrobial activity was observed. The reason for also synthesizing the most concentrated heterophase in brookite is motivated by the literature [67], according to which the mixed phases could present more evident antimicrobial and/or antibacterial activity. In all three samples, pristine TiO_2_, TiO_2_ MPs/SM (anatase/brookite), and TiO_2_ MPs/SM (brookite/anatase), the micrometric size of the particles and the functionalization by physisorption could provide no activated TiO_2_ particles. The carbonyl groups of the terpenoids are physically adsorbed (see Table 5 for Z-potential and TGA values) and not chemically anchored to the TiO_2_ microparticles (see FTIR that reveals the presence of organic functional groups but not the formation of a new chemical bond, as demonstrated in the case of CaCO_3_ MPs/SM). Functionalization that occurs through chemical bonding might be able to intrinsically modify the TiO_2_ particle, thus making it reactive towards diversified bacterial strains. In fact, there are works in the literature that demonstrate how particles of 600 nm (sizes comparable to those of our particles) [68] and particles of 2000 nm [69] (far larger than ours), with different syntheses, have been chemically functionalized and exhibit high antimicrobial and antibacterial activity (regardless of micrometric dimensions). In light of these data, future studies could concern the UV photoactivation of TiO_2_ during the green synthesis in SM aqueous extracts [70], performed at low temperatures (ranging from 40 to 60 °C) and atmospheric pressure.

**Scheme 2:**
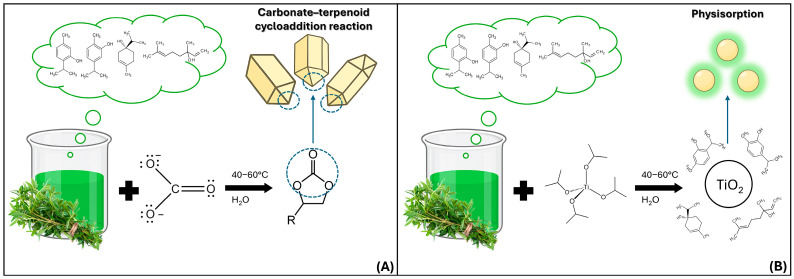
Different functionalization reactions: (**A**) carbonate cycloaddition to aromatic rings and/or allene molecules, (**B**) physical adsorption interactions.

The opposite happens working with chemically inert and large-sized CaCO_3_ MPs/SM prismatic particles. Their toxicity towards bacteria derives from the fact that the functionalization occurs through a new established chemical bonding (see FTIR data) which leads to the formation of cyclic carbonates (Scheme 2), anchored on the aromatic ring of terpenoid molecules [71]. Therefore, CaCO_3_ particles and terpenoids merge intimately until they become a negatively charged single structure (see Table 5 for Z-potential and acidic titration values), having a very angular prismatic morphology (see SEM on Figure 3) and an increased porosity (Table 6 for BET analysis). This is not observed for pristine CaCO_3_ microparticles, which are prismatic but much smoother and less angular (as shown by the SEM micrographs in the Supplementary Information), and this happens when the cycloaddition reaction does not occur.

Cyclic carbonates are green chemistry building blocks often synthesized from bio-based epoxides, such as those derived from terpenes and terpenoids, and the greenhouse gas carbon dioxide (CO_2_) through a cycloaddition reaction [72]. Terpene-derived cyclic carbonates are explored as bio-based cyclic solvents and are useful for antimicrobial in vitro tests because of their ability to dissolve both organic and inorganic compounds, providing testing of various compounds by the disk diffusion, agar dilution, or broth dilution assays, which are standard techniques for assessing antimicrobial activity [73].

## 3. Materials and Methods

In accordance with what was reported above for the results and their discussion, the experimental part will also follow the same order of presentation, i.e., materials/measurement procedures for the synthesis and characterization of microparticles and natural extracts from SM; in the second part the materials/measurement procedures for the characterization of the antibacterial, antimicrobial, anti-oxidant, and anti-biofilm properties of microparticles functionalized with natural extracts of SM will be described, respectively.

### 3.1. Plant Materials and Chemicals/Reagents

SM plants (*Satureja montana* L., Lamiaceae, commonly known as winter savory) were collected from Orto Botanico di Roma Sapienza University (Italy) during the flowering phase (on July 2024), where the term of harvest had a significant effect on the chemical content of essential oils and natural extracts in SM [74]. Each plant during the sampling step was hand-harvested into Ziploc storage containers and transported to the laboratory. Leaves were manually removed and left to dry at room temperature and under pressure for two weeks until extraction.

All chemical reagents are of analytical grade and purchased from Merck (Germany), including titanium tetra isopropoxide (TTIP, C_12_H_28_O_4_Ti, 97%), ethanol (C_2_H_5_OH, 96%), n-hexane (C_6_H_14_, 98.5%), and the following GC-MS standards: α-humulene (C_15_H_24_, 90%), α-phellandrene (C_10_H_16_, 85%), α-pinene (C_10_H_16_, 98%), α-terpinolene (C_10_H_16_, 95%), aromadendrene oxide 2 (C_15_H_24_O, 95%), β-caryophyllene (C_15_H_24_, 80%), β-myrcene (C_10_H_16_, 90%), β-pinene (C_10_H_16_, 99%), carvacrol (C_10_H_14_O, 98%), carvacrol methyl ether (C_11_H_16_O, 90%), caryophyllene oxide (C_15_H_24_O, 98%), *cis*-α-bisabolene (C_15_H_24_, 93%), *cis*-sabinene hydrate (C_10_H_18_O, 97%), D-carvone (C_10_H_14_O, 98%), geraniol (C_10_H_18_O, 98%), (R)-(+)-limonene (C_10_H_16_, 97%), *γ*-terpinene (C_10_H_16_, 95%), L-linalool (C_10_H_18_O, 95%), *para*-cymene (C_10_H_14_, 99%), sabinene (C_10_H_16_, 75%), (+)-terpinen-4-ol (C_10_H_18_O, 98%), and thymol (C_10_H_14_O, 99%). Other used chemicals and solvents were of the highest analytical grade, as perdeuterated fluoranthene (Chemical Research 2000 S.r.l., Milan, Italy) and NaCl (Merck, Germany). Bidistilled water was obtained with the Millipore system (Milli-Q^®^ EQ 7000), and all aqueous and/or hydroalcoholic solutions were freshly prepared every day, to carry out all the measurements.

### 3.2. Preparation of TiO_2_ Microparticles in SM Natural Extracts (TiO_2_ MPs/SM)

SM natural extracts were obtained by drying the leaves of the plants (3 plants). The latter were harvested, dried, and subsequently ground using a mortar. To prepare the extract, 2 g of the pulverized leaves were immersed in 60 mL of bidistilled water and heated to 40 °C under continuous stirring for 1 h. The resulting extract was filtered, cooled to room temperature, and stored in a dark glass bottle at 4 °C. This protocol agrees with the literature [75], but we modified it for the aqueous (and not organic) solvent and for the thermal treatment at lower temperature.

**Scheme 3:**
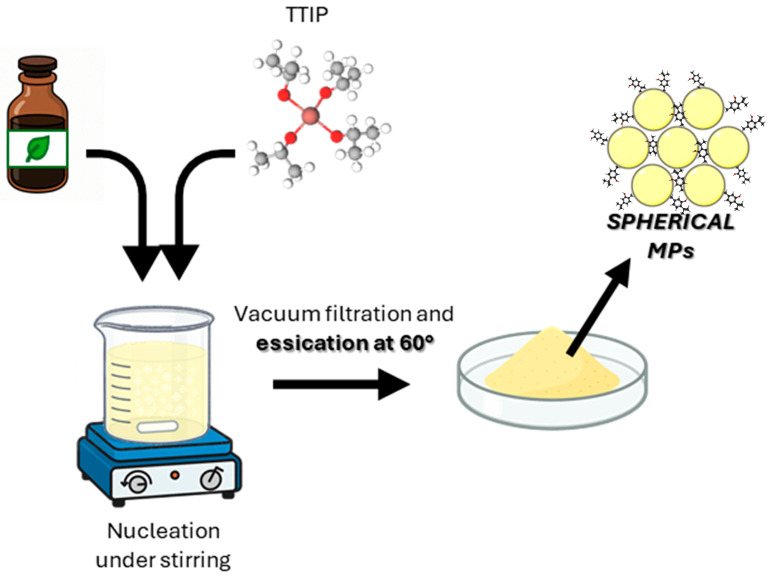
Synthesis protocol of TiO_2_ MPs/SM.

TiO_2_ particles were synthesized starting from titanium isopropoxide (TTIP, used here as titanium oxides precursor, see Scheme 3) in ethanol as a working medium, according to the literature [76]. Briefly, 5 mL of TTIP was dissolved in 5 mL of ethanol solution and 2 mL of aqueous SM extract (added drop by drop into the reaction environment), under continuous stirring for 3 h at room temperature (RT). The solid was vacuum filtered, washed thoroughly with 250 mL of double-distilled water, and finally dried in an oven at 60 °C (without calcination step, which is known to occur at temperatures well above that used in this study). The resulting TiO_2_ MPs/SM were collected and processed with further characterization measurements.

### 3.3. Preparation of CaCO_3_ Microparticles in SM Natural Extracts (CaCO_3_ MPs/SM)

**Scheme 4:**
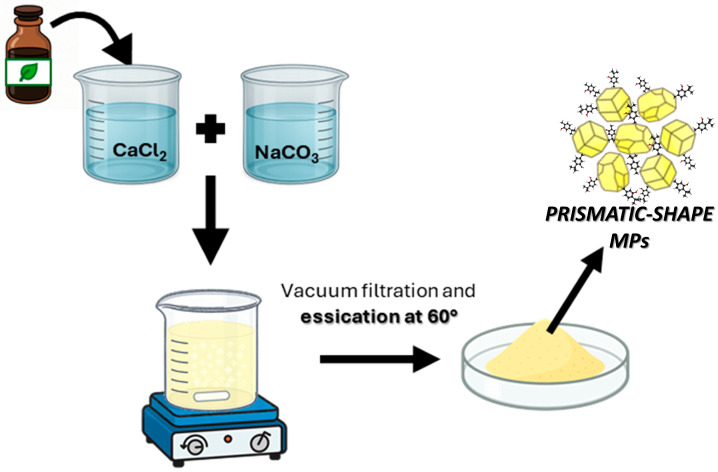
Synthesis protocol of CaCO_3_ MPs/SM.

SM natural extracts were obtained according to the previous paragraph. CaCO_3_ particles were synthesized starting from calcium chloride (CaCl_2_) and sodium carbonate (Na_2_CO_3_) used here as a precursor (see Scheme 4), according to the literature [77,78]. Briefly, 200 mL of 50 mM CaCl_2_ and 50 mL of aqueous SM extract were mixed together under magnetic stirring at 400 rpm for 5 min (at R.T.). Then, 250 mL of 50 mM Na_2_CO_3_ was added to the previous solution, which was left under magnetic stirring at 400 rpm for 15 min (at R.T.). The solid was vacuum filtered, washed thoroughly with 250 mL of double-distilled water, and finally dried in an oven at 60 °C. The resulting CaCO_3_ MPs/SM were collected and processed with further characterization measurements.

### 3.4. TiO_2_ MPs/SM) and (CaCO_3_ MPs/SM) Characterization Study

In this paragraph, the order of presentation of the measuring apparatus and experimental procedures performed will follow the one adopted previously in the text, for the presentation of the experimental results.

#### 3.4.1. SEM/EDX

The method is the same for both CaCO_3_ microparticles and TiO_2_ microparticles. An ethanol-based (100%) suspension containing a small amount of microparticles was prepared in a glass tube and exposed to one hour of sonication. Upon completion, 10 µL of the suspension was rapidly drop-cast onto 200-mesh copper grids coated with Formvar film (Ted Pella, Tustin, CA, USA). These grids were then set atop absorbent paper inside a Petri dish, covered, and left to dry under ambient conditions. Once the grids were fully dried, they were mounted onto aluminum stubs using carbon tape and introduced into a sputter coater (Emitec K550, Emitech Corato, Italy), where they were coated with platinum at a current of 15 mA for 1.5 min. This sputtering process was conducted twice to ensure uniform coating. Sputter coating was necessary to ensure image quality at the higher magnifications required for a morphological characterization and a reliable EDX analysis; the presence of a significant reduction in surface conductivity strongly suggested the presence of organic matter on the NPs’ surface (as stated from other analytical methods). We did not observe surface charging in previous observations of NPs whose surface was without organic matter.

For the scanning electron microscopy and image analysis, samples were examined using a variable-pressure scanning electron microscope (VP-SEM), specifically the Hitachi SU3500 model (Hitachi, Tokyo, Japan), operated under high vacuum conditions at an accelerating voltage of 15–20 kV [79,80,81]. This SEM system was equipped with dual energy-dispersive X-ray spectroscopy (dEDS) detectors (Bruker XFlash^®^ 6|60), enabling simultaneous multimodal imaging and spatially resolved chemical mapping. This advanced analytical capability was particularly effective for investigating biological surfaces in their native state. The XFlash^®^ 6|60 was well suited for nanoanalytical applications involving materials with relatively low X-ray emission [76,77]. For the image analysis, the SEM micrographs were collected using Hitachi Map 3D software, version 8.2 (Digital Surf, Besançon, France) [82,83].

#### 3.4.2. XRD

Powder X-ray diffraction (PXRD) analyses were carried out using a Rigaku SmartLab SE powder diffractometer (Rigaku, Tokyo, Japan), operating with a Cu Kα radiation anode at 40 kV and 30 mA, and equipped with a Rigaku D/teX Ultra 250 silicon strip detector. As the instrument is designed for powder analysis, samples were analyzed directly, without any additional preparation. To improve peak resolution in the high-angle region and to detect weaker diffraction signals, a second set of PXRD measurements was acquired using a D8 Advance diffractometer (Bruker, Billerica, MA, USA) equipped with a Mo Kα radiation source. The use of a shorter X-ray wavelength enabled the detection of additional reflections that were not clearly resolved under Cu Kα irradiation.

#### 3.4.3. Raman Spectroscopy

Samples were analyzed using a micro-Raman system equipped with a Cobolt 08-DPL 532 nm solid-state laser (Cobolt AB, Solna, Sweden), coupled with an HRS500 spectrometer (Teledyne Princeton Instruments, Trenton, NJ, USA) with a 50 cm focal length and a 100× objective (NA = 0.9, model MPLFLN100XBD, Olympus Corporation, Tokyo, Japan), ensuring high spatial resolution. All spectra were collected directly on the microparticle powders without specific sample preparation.

#### 3.4.4. FTIR Spectroscopy

The Fourier transform infrared spectroscopy (FTIR) measurements were performed in attenuated total reflection (ATR) mode. A Nicolet Summit FTIR spectrometer (Thermo Fisher Scientific Inc., Waltham, MA, USA), equipped with an Everest™ Diamond ATR accessory (Thermo Fisher Scientific Inc., Waltham, MA, USA), was used. Spectra were acquired with the following parameters: 32 scans per sample, a resolution of 8 cm^−1^, and a spectral range of 4000–600 cm^−1^. As the analysis was performed using FTIR in ATR mode, the powdered samples were placed directly in contact with the diamond crystal, without sample treatments.

#### 3.4.5. Zeta-Potential, DLS, and MALS

The particle size distribution (DLS) and the Z-potential (ξf/mV) measurements were carried out by using Zetasizer Nano ZS equipment (Malvern, UK), according to our previous paper [84]. This apparatus is equipped with a back-scattering detection mode (with an angle of 173°), and a He-Ne laser with a wavelength of 633 nm (laser Doppler velocimetry). Samples were sonicated in ultra-pure filtered water (using a Teflon filter with a pore diameter of 100 nm) for 30 min, diluting the original dispersion (1 mg/mL) to the final concentration of 0.001 mg/mL. For the hydrodynamic diameter of CaCO_3_ MPs/SM prismatic particles, a MALS (multi-angle static sight scattering) detector was applied. MALS examines the angular dependence of the time-averaged scattering intensity to determine the mass-averaged root mean square radius R g (also known as ‘radius of gyration’) from 10 nm to several hundred nanometers, independent of shape, as described in classical light scattering theory. If the sample does not conform to a sphere, the R_g_ can be determined to as much as 1000 nm by a DAWN ™ 18-angle MALS detector [85].

#### 3.4.6. TGA

Thermogravimetric analysis/TGA was carried out by a Q600 thermogravimetric analyzer (TA Instruments-Waters, New Castle, DE, USA). Samples (~20 mg) were put in a platinum crucible and heated from 30 °C to 700 °C, with a rate of 10 °C/min, under nitrogen purge gas. Measurement values are reported in Table 5 as (mean ± SD).

#### 3.4.7. BET Analysis

The specific surface area (Brunauer–Emmett–Teller, BET method) and total pore volume (as originally proposed by Gurvitsch [86]) were determined by nitrogen adsorption/desorption at −196 °C using a 3Flex 3500 Micromeritics analyzer, after outgassing the samples at 200 °C for 2 h, according to our previous work [87]. Briefly, the pore size distribution was evaluated using the Barrett–Joyner–Halenda (BJH) method, from the adsorption isotherm [88]. The uncertainty in specific surface area measurements was ±0.5 m^2^/g. The porosity in the 0.0037–150 μm range (mesoporosity) and the pore size distribution were also determined with the same instrument. The mesoporosity, together with total open porosity, allowed estimation of the microporosity (pores with radius ≤ 0.0037 μm), according to the pore space classification proposed in previous literature [89,90].

#### 3.4.8. GC-MS Analysis of SM Extract: Molecular Composition

The chemical composition of the aqueous extracts obtained from SM leaves was investigated using an untargeted approach based on gas chromatography coupled with high-resolution Orbitrap mass spectrometry (GC-HRMS). The analyses were performed on a Thermo Scientific™ system operated in full scan mode, with a resolving power of up to 50,000 FWHM at *m*/*z* 272 and mass accuracy below 1 ppm.

Headspace solid-phase microextraction (HS-SPME) was performed using a Tri Plus RSH autosampler (Thermo Fisher Scientific Inc., Waltham, MA, USA) coupled with an 80 µm thick, 10 mm long multi-adsorbent DVB/CarbonWR/PDMS fiber (Supelco, Merck KGaA, Darmstadt, Germany). The fiber was initially conditioned at 280 °C for 60 min. For all samples, the fiber was pre-conditioned for 5 min at 270 °C and post-conditioned for 3 min at 270 °C after injection. Headspace extraction of samples was carried out in 20 mL vials containing 2 g of NaCl, previously dried at 300 °C for 4 h. To these vials, 10 mL of sample was added, spiked with 20 ng of perdeuterated fluoranthene (Chemical Research 2000 S.r.l., Milan, Italy) as an internal standard. Each vial was conditioned at 70 °C for 25 min prior to fiber sampling. The SPME fiber was exposed to the headspace vapors for 10 min under agitation at 5 revolutions/minute. The SPME needle penetrated the vial by 40 mm, ensuring the full 10 mm length of the fiber was exposed. Subsequently, the fiber was desorbed for 5 min at 270 °C in a split/splitless injector in split mode (1:50).

Chromatographic separation was performed using an Rxi-5Sil MS column (30 m × 0.25 mm I.D. × 0.25 µm film thickness; Restek Corporation, Bellefonte, PA, USA), operated under a programmed temperature gradient. The carrier gas was helium 5.5 (Nippon Gases Italia S.r.l., Milan, Italy) at a constant flow rate of 1 mL/min. The GC oven temperature program started at 40 °C, held for 5 min, then increased at 6 °C/min to 150 °C, and subsequently at 15 °C/min to 280 °C, with a final isothermal hold of 3 min.

A dedicated analytical batch was designed to ensure reproducibility and background control. This batch included procedural blanks—Milli-Q water and filtered water—processed and analyzed under identical conditions as the SM extract samples. These blanks were used for background subtraction during data processing. Additionally, the batch included a five-point calibration curve of a terpene standard mixture at concentrations of 0.08, 0.20, 1.00, 2.00, and 2.01 ng/µL, to each point of which 20 ng of perdeuterated fluoranthene internal standard was added, enabling semi-quantitative analysis of the identified compounds.

Raw data were processed using Compound Discoverer™ 3.3.3.200 software following a two-step deconvolution workflow. In the first step, all features were extracted and aligned across the dataset, with blank subtraction applied to remove procedural background. In the second step, the same dataset was reprocessed to quantify the analytes by comparing the response intensities against the terpene standard mixture, enabling a semi-quantitative estimation of compound abundance.

Peak detection parameters were configured to ensure robust compound annotation. The mass tolerance was set at 5 ppm, the minimum spectral signal-to-noise ratio at 10, and the minimum chromatographic peak S/N threshold at 3. A total ion current (TIC) threshold of 1,000,000 was applied based on signal intensities observed in real samples. The ion overlap for spectral matching was fixed at 98%, and high-resolution filtering (HRF) scoring was enabled. For compound identification via spectral libraries, both the similarity index (SI) and reverse similarity index (RSI) thresholds were set at 600.

All detected features were filtered for reproducibility across replicates, retention time consistency, and spectral match quality. Confidently identified compounds were grouped into major chemical families, including monoterpenes, sesquiterpenes, diterpenes, phenolic compounds, and long-chain fatty acid derivatives. This comprehensive VOC fingerprint of SM provided the chemical basis for interpreting the role of the molecular activators/phase controllers during the inorganic particles synthesis.

### 3.5. Experimental Biological Testing and Sampling

To evaluate the potential antimicrobial properties of TiO_2_ and CaCO_3_ MPs, 500 µL of each solution at the previously specified concentrations (0.2, 2, 4, 10, 20, 50 mg/mL) were aseptically dispensed onto Petri dishes containing 25 mL of culture medium (plate count agar, Liofilchem). The solution was evenly distributed across the agar surface under sterile conditions to ensure homogeneous coverage, and plates were subsequently used for the sampling phase. The sampling was performed using the passive sedimentation method, which measures the amount of microorganisms that settle on surfaces by exposing settle plates to air for a defined period of time [91]. In detail, rather standard 9 cm Petri dishes were used to collect the biological particles as sediments, randomly positioned in a public indoor area of 25 m^2^, according to the 1/1/1 scheme (for 1 h, 1 m above the floor, and about 1 m away from walls and major obstacles) [92]. For each treatment, six opened MP-treated Petri dishes were used, and six untreated plates were used as controls (CT). At the end of the exposure period, the plates were incubated at a temperature of 25 °C for 48 h. Bacterial colonies were manually counted by placing each Petri dish on a gridded plate and enumerating colonies within each cell, and the results are expressed as CFUs per plate (CFU/plate).

#### Statistical Analyses

Statistical analyses were performed using GraphPad Prism 10.1.2 (GraphPad Software Inc., San Diego, CA, USA). Data normality was verified with the Shapiro–Wilk test (*p* < 0.05), allowing parametric analysis by ANOVA followed by Tukey’s post hoc test. Significance was set at *p* < 0.05.

## 4. Conclusions

TiO_2_/A and CaCO_3_/C microparticles, modified with the aqueous extract of the SM plant in the main form of anatase (A) and calcite (C), were synthesized, characterized, and tested for their antimicrobial activity. All the TiO_2_ MPs/SM samples and the corresponding pristine microparticles did not show significant antimicrobial activity because the terpenoid functional groups were not chemically anchored to microparticle surfaces. What occurs between the TiO_2_ microparticles and the terpenoids extracted by SM in water is not chemisorption but physisorption. This makes the microparticles inactive, unlike other microparticles (even larger ones), which, as stated in the literature, have significant antimicrobial activity. Future studies will involve the same low-temperature synthetic route in aqueous SM extract, UV-photochemically activating titanium dioxide to promote the chemical anchoring of terpenoids on microparticle surfaces.

Instead, CaCO_3_ MPs/SM exhibit strong and dose-dependent antimicrobial activities, achieving near-complete inhibition at 50 mg/mL. This effect is mainly due to the chemical (and not physical) functionalization of calcite microparticles, which occurs according to a well-known cycloaddition and/or 2,2-Diels–Alder reaction. These known reaction mechanisms result in cyclic carbonates, chemically anchored to the aromatic rings of terpenoids or even to molecular allenes (the latter always forming part of the structure of terpenes, as natural substances).

Future study will be carried out directly in samples of bacterial cultures treated with CaCO_3_ MPs/SM ready to be used, through variable-pressure electron microscopy (VP-SEM), where the bacterial strains will be grown directly on the SEM aluminum stubs.

## Figures and Tables

**Table 1:** Raman and FTIR band assignments of TiO_2_ MPs/SM.

Band (cm^−1^)	Assignment	References
	RAMAN SPECTROSCOPY	
146	E_g_ mode (anatase)	[32,33,34]
219–279	E_g_ (anatase) B_1g_ + B_2g_ mode (brookite)	[32,33,34,35]
411	B_1g_ mode (anatase)	[32,33,34]
517	A_1g_ + B_1g_ mode (anatase)	[32,33,34]
623	E_g_ mode (anatase)	[32,33,34]
~874	νC–OH νC–C δCH/CH_2_	[36,37,38,39,40,41,42,43,44,45]
~1627	νC=O νC=C δO–H	[36,37,38,39,40,41,42,43,44]
~3352	νO–H	[46]
	FTIR SPECTROSCOPY	
3400	νO–H	[47,48,49,50,51]
1630	δO–H νC=O νC=C	[47,48,49,50,51]
2974	ν_as_–CH_3_	[50,51]
2893	ν_s_–CH_2_	[50,51]
1454, 1395	δ_as/s_–CH_3_ δ_as/s_–CH_2_	[50,51]
1268	νC–O νC–C	[50,51]
1051	νC–O νC–C ring vibration	[50,51]
800–600	ν + δ Ti–O–Ti	[52,53]

Vibrational band assignments follow conventional spectroscopic notation: ν = stretching; δ = bending; s = symmetric; as = asymmetric.

**Table 2:** Raman and FTIR band assignments of CaCO_3_ MPs/SM.

Band (cm^−1^)	Assignment	References
RAMAN SPECTROSCOPY		
248	E_g_ lattice mode (calcite) τ + δ aromatic ring	[55,56]
320	τ aromatic ring	[36,37,38,39,40,44]
412	δ_op_ + τ aromatic ring	[36,37,38,39,40,44]
507	δ_op_ + τ aromatic ring	[36,37,38,39,40,44]
626	δ_op_ + τ aromatic ring	[36,37,38,39,40,44]
718	E_g_ mode (δ_ip_CO_3_^2−^)	[55,56]
746	νC–C δC–O δ aromatic ring	[36,37,38,39,40,44]
890	νC–OH νC–C δCH/CH_2_	[36,37,38,39,40,44]
1009	δ_ip_CH νC–O δ aromatic skeletal	[37,38,39,40,41,42,43,44]
1092	A_1g_ mode (ν_s_CO_3_^2−^)	[55,56]
1180	δCH νC–O δ aromatic skeletal	[36,37,38,39,40,41,42,43,44,45]
1339	δCH_3_ (isopropyl) δCH_2_	[36,37,38,39,40,41,42,43,44,45]
FTIR-ATR SPECTROSCOPY		
3600–3000	νO–H	[48,49,50,51]
2951	ν_as_–CH_3_	[47,48,49,50,51]
2922	ν_as_–CH_2_	[47,48,49,50,51]
2856	ν_s_–CH_2_	[47,48,49,50,51]
1710	νC=O	[47,48,49,50,51]
1637	δO–H νC = O νC = C	[47,48,49,50,51]
1404	ν_as_CO_3_^2−^	[57,58]
1231	νC–O νC–C δ aromatic skeletal	[47,48,49,50,51]
1070	νC–O νC–C δ aromatic skeletal	[47,48,49,50,51]
870	δ_op_CO_3_^2−^	[57,58]
705	δ_ip_CO_3_^2−^	[57,58]

Vibrational band assignments follow conventional spectroscopic notation: ν = stretching; δ = bending; τ = twisting; s = symmetric; as = asymmetric; ip = in-plane; op = out-of-plane.

**Table 3:** Semi-quantitative concentrations of VOCs identified by targeted GC-MS (n.d. = not detected).

Compound	Concentration [ng/mL]
α-thujone	282
α-pinene	n.d.
camphene	n.d.
sabinene	1054
β-pinene	n.d.
α-phellandrene	264
α-terpinene	3814
para-cymene	157
(R)-(+)-limonene	535
α-terpinolene	284
γ-terpinene	882
terpinen 4-ol	10,305
carvacrol methyl ether	23.4
L-carvone	n.d.
thymoquinone	8271
geraniol	n.d.
thymol	63,906
carvacrol	5669
β-caryophyllene	n.d.
α-humulene	4.0
caryophyllene oxide	135
cis-α-bisabolene (levomenol)	4.7
L-linalool	1425
aromadendrene oxide 2	n.d.

**Table 4:** Mean ± SD (*n* = 6) of CFU/plate following passive sedimentation method under different TiO_2_ and CaCO_3_ MPs treatments. CT: untreated control. Statistical significance relative to the untreated control is indicated by asterisks: ** *p* < 0.005; *** *p* < 0.001; **** *p* < 0.0001.

Treatment	Concentration (mg/mL)	CFU/Plate	MPs Size _SEM (nm/µm)
Titanium Oxide			
CT	-	86.00 ± 3.60	
TiO_2_ MPs/SM	0.2	184.00 ± 10.58	Diameters ranging from 160 nm to 1.3 µm, with a prevalence of elements with diameters around 400–600 nm
	2	142.40 ± 29.70	
	4	274.00 ± 47.62 ***	
	10	1132.00 ± 46.13 ****	
	20	>2500	
	50	>2500	
TiO_2_ MPs (pristine)	0.2	1776.00 ± 166.40 ****	Diameters about 200 nm
	2	2452.00 ± 22.27 ****	
	4	>2500	
	10	>2500	
	20	>2500	
	50	>2500	
Calcium Carbonate			
CT	-	106.00 ± 13.53	
CaCO_3_ MPs/SM	0.2	102.00 ± 2.00	CaCO_3_ particles have prismatic shape, sharp edges, and variable size, with an average of 1 µm × 1 µm × 1 µm
	2	97.00 ± 1.73	
	4	75.33 ± 16.04 **	
	10	55.67 ± 9.02 ****	
	20	6.33 ± 4.04 ****	
	50	1.00 ± 1.00 ****	
CaCO_3_ MPs (pristine)	0.2	111.7 ± 7.23	Diameters about 800 nm
	2	108.3 ± 9.07	
	4	101.00 ± 7.93	
	10	100.70 ± 7.02	
	20	100.70 ± 10.07	
	50	96.00 ± 5.29	

**Table 5:** Z-potential, DLS/MALS analysis, acidic site quantification, and weight loss (%) by TGA.

All Synthetized Samples	Z-Potential (ξ, mV)	MALS * Hydrodynamic Diameter (µm)	Acidic Sites ^(a)^ (nmol/mg)	Weight Loss % (TGA)
TiO_2_ MPs/SM (Anatase)	−8.92	1.57 (by DLS) spherical particles	14.66 ± 0.97 (FTIR signal for C(=O) carbonyls)	1.41 ± 0.40
TiO_2_ MPs/SM (Brookite/Anatase) heterophase	−6.07	2.03 (by DLS) spherical particles	9.98 ± 0.45 (FTIR signal for C(=O) carbonyls)	0.95 ± 0.23
TiO_2_ MPs (pristine) ^(b)^	------	0.31 (by DLS) spherical particles	------	------
CaCO_3_ MPs/SM (Calcite)	−25.14	2.83 (by MALS) prismatic particles	41.31 ± 1.23 (FTIR signal for C(=O) O-H carboxyls)	3.97 ± 0.34
CaCO_3_ MPs (pristine) ^(b)^	------	0.72 nm (by MALS) prismatic particles	------	------

*: DLS is most accurate for spherical particles or particles with small deviations from sphericity. For highly asymmetric or complex shapes, other techniques like multi-angle light scattering (MALS) might be more appropriate (described in Section 3 Materials and Methods). ^(a)^: These reactive functional groups were also quantified through the volumetric titration of the acidic sites, as reported in literature [19] (mean ± SD) ^(b)^: In the table for the pristine particles, the data on Z-potential, the concentration of acidic groups, and the TGA analysis of the % weight loss of oxygenated groups are missing; this is because they are not functionalized with SM; therefore there are no functional groups anchored on the surface of the pristine particles. The hydrodynamic diameter for TiO_2_ particles was measured with DLS since they are spherical (as confirmed by SEM), while for CaCO_3_ particles, the hydrodynamic diameter was measured with MALS since they are non-spherical in shape (as confirmed by SEM).

**Table 6:** BET analysis and porosity data. Antimicrobial results summary.

Samples	Surface Area (m^2^/g)	Pore Volume (cm^3^/g)	Pore Size (nm)	Antimicrobial Activity (Figure 7 and Table 4)
TiO_2_ MPs/SM (Anatase)	1.20	0.0023	560	Not detected
CaCO_3_ MPs/SM (porous calcite)	20.0	0.0383	1000	Detected
TiO_2_ MPs/SM (Brookite/Anatase) heterophase	0.84	0.0011	650	Not detected
TiO_2_ (pristine)	13.3	0.046	180	Not detected
CaCO_3_ (pristine)	100	0.581	650	Not detected

## Data Availability

Data are contained within the article.

## Supplementary Materials

The following supporting information can be downloaded at: https://www.mdpi.com/article/10.3390/molecules30204138/s1, Table S1. Nomenclature and abbreviations of all materials cited in the text. Table S2. List of compounds identified in the *Satureja montana* aqueous extract by untargeted GC-HRMS. The table includes only features with high-confidence identifications (Total Score > 90) and background-subtracted signals. For each compound, the calculated molecular weight, retention time (RT), reference *m*/*z*, average total ion current (TIC), molecular formula (as matched with NIST library), theoretical and observed molecular masses, spectral matching scores (Total Score, HRF Score, SI), and group-specific peak areas are reported. Peak areas correspond to injections of *Satureja montana* (SM) and *Satureja montana* after treatment and filtration (SM-TF). The peak rating column indicates the library match quality on a scale from 1 to 10. Text S1. Additional characterization for TiO_2_ MPs/SM, CaCO_3_ MPs/SM, and pristine particles (i.e., TiO_2_ and CaCO_3_ without SM). Figure S1. Raman spectrum of the organic activators/phase controllers’ fingerprint into TiO_2_ MPs/SM sample (where Anatase is the major component, as reported in the full text). Raman spectra were collected at different points of the TiO_2_ MPs/SM sample. The spectra were normalized based on the integration time used (120 s for points 2 and 3, and 60 s for points 4, 5, and 6). Figure S2. TiO_2_ MPs/SM: (A) micrograph and (B) EDX analysis. CaCO_3_ MPs/SM: (C) micrograph and (D) EDX analysis for calcite. Figure S3. XRD of TiO_2_ MPs/SM as brookite polymorphic crystalline form (i.e., mixed phase brookite/anatase, having brookite as the major component). Figure S4. Raman spectrum of TiO_2_ MPs/SM as heterogeneous phase brookite/anatase, having brookite as the major component. Table S3. Raman band assignments of TiO_2_ MPs/SM as heterogeneous phase brookite/anatase. Scheme S1. Comparison between conventional high-temperature calcination synthesis and the green route developed in this work for TiO_2_ production. Figure S5. SE-micrographs of pristine particles. (A) Pristine TiO_2_ Anatase particles cluster is shown. Dotted circles enclose particles with a diameter of about 200 nm, magnification 20K. Bar 2 µm. (B) Pristine CaCO_3_ Calcite particles present defects (square) and the edges are rounded and not sharp (dotted ovals), having dimensions of about 800 nm, magnification 20K. Bar 2 µm. Scheme S2. Synthesis routes for pristine TiO_2_ and CaCO_3_. Top: anatase TiO_2_ obtained by sol–gel hydrolysis of titanium(IV) isopropoxide (TTIP); bottom: calcite CaCO_3_ produced by precipitation from CaCl_2_ and NaCO_3_. Scheme S3. Optimization synthesis protocol to increase the phase controllers/molecular activators effect/efficiency on several kind of inorganic particles, to improve their antimicrobial and antibacterial performances against selective microorganism strains of interest in numerous clinical-medical, pharmacological, environmental, food, agriculture and Cultural Heritage application fields. References are cited in the Supplementary Materials [93,94,95,96,97,98,99,100,101,102,103,104].
