# Supplementary material for: TiO2 and CaCO3 Microparticles Produced in Aqueous Extracts from Satureja montana: Synthesis, Characterization, and Preliminary Antimicrobial Test
Source: Molecules. 2025 Oct 20;30(20):4138. doi: 10.3390/molecules30204138 (PMC12566081; doi:10.3390/molecules30204138)
Supplement: Supplementary file 1 [file molecules-30-04138-s001.zip › molecules-3831393-SM.pdf]

molecules

MDPI

Article

TiO_2 and $CaCO_3$ Microparticles Produced in Aqueous Extracts from *Satureja montana*: Synthesis, Characterization, and Preliminary Antimicrobial Test

Federica Valentini [1,*], Irene Angela Colasanti [1,2], Camilla Zaratti [1,2], Dumitrita Filimon [1], Andrea Macchia [3], Anna Neri [4], Michela Relucenti [5], Massimo Reverberi [6], Ivo Allegrini [7], Ettore Guerriero [8], Marina Cerasa [8], Marta De Luca [9], Francesca Santangeli [9], Roberto Braglia [10], Francesco Scuderi [10], Lorenza Rugnini [10], Roberta Ranaldi [10], Roberto De Meis [10] and Antonella Canini [10]

1. Sciences and Chemical Technologies Department, Tor Vergata University, Via della Ricerca Scientifica 1, 00133 Rome, Italy; ireneangela.colasanti@students.uniroma2.eu (I.A.C.); camilla.zaratti@students.uniroma2.eu (C.Z.); filimon.1768827@studenti.uniroma1.it (D.F.)
2. YOCOCU APS, Via Torquato Tasso 108, 00185 Rome, Italy
3. Department of Biology, Ecology and Earth Sciences (DiBEST), University of Calabria, Via Pietro Bucci, 87036 Arcavacata, Italy; aps@yococu.com
4. Department of Biomedicine and Prevention, Tor Vergata University, Viale Montpellier 1, 00133 Rome, Italy; anna.neri@uniroma2.it
5. Department of Anatomical, Histological, Forensic and Orthopaedic Sciences, Sapienza University of Rome, Via Alfonso Borelli 50, 00161 Rome, Italy; michela.relucenti@uniroma1.it
6. Department of Environmental Biology, Sapienza University of Rome, Piazzale Aldo Moro 5, 00185 Rome, Italy; massimo.reverberi@uniroma1.it
7. Envint Srl, Via Paradiso 65a, Montopoli di Sabina, 02034 Rieti, Italy; ivo.allegrini@tiscali.it
8. Institute of Atmospheric Pollution Research (IIA), National Research Council (CNR), Strada Provinciale 35d, 9, 00010 Montelibretti, Italy; ettore.guerriero@cnr.it (E.G.); marina.cerasa@cnr.it (M.C.)
9. Department of Physics, Sapienza University of Rome, Piazzale Aldo Moro 2, 00185 Rome, Italy; marta.deluca@uniroma1.it (M.D.L.); francesca.santangeli@uniroma1.it (F.S.)
10. Department of Biology, Tor Vergata University of Rome, Via della Ricerca Scientifica 1, 00133 Rome, Italy; roberto.braglia@uniroma2.it (R.B.); francesco.scuderi@uniroma2.it (F.S.); lorenza.rugnini@uniroma2.it (L.R.); ranaldiroberta@gmail.com (R.R.); roberto.demeis@gmail.com (R.D.M.); canini@uniroma2.it (A.C.)

* Correspondence: federicavalentini.chem@gmail.com

Supplementary Materials

Table S1. Nomenclature and abbreviations of all materials cited in the text.

Nomenclature	Acronyms/abbreviations
Titanium Oxide	TiO_2 MPs/SM
Calcium Carbonate	$CaCO_3$ MPs/SM
Brookite/Anatase heterophase	Brookite/Anatase MPs/SM
Microparticles	MPs
Nanoparticles	NPs
Satureja montana	SM
Natural Plant Extracts	NPEs
Essential Oils	EOs
Nano-Emulsions	NEs
Volatile Organic Compounds	VOCs
Radical Oxygenated Species	ROS

Table S2. List of compounds identified in the *Satureja montana* aqueous extract by untargeted GC-HRMS. The table includes only features with high-confidence identifications (Total Score > 90) and background-subtracted signals. For each compound, the calculated molecular weight, retention time (RT), reference m/z, average total ion current (TIC), molecular formula (as matched with NIST library), theoretical and observed molecular masses, spectral matching scores (Total Score, HRF Score, SI), and group-specific peak areas are reported. Peak areas correspond to injections of *Satureja montana* (SM) and *Satureja montana* after treatment and filtration (SM-TF). The Peak Rating column indicates the library match quality on a scale from 1 to 10.

Name	RT [min]	Avg TIC	NIST Lib Hit Formula	NIST Theo. Mol. Mass	NIST Observed Mol. Mass	Total Score	HRF Score	SI	SM	Peak Rating (Max.)
(-)-10-Camphorsulfonyl chloride	24.32	7832076	C10 H15 Cl O3 S	250.0425		92	99.6	607	1690690	10
(-)-R-Phenethanamine, 1-methyl-N-vanillyl-	24.37	346648	C17 H21 N O2	271.1567		95.3	100	765	157065	7.5
(+)-2-Bornanone	16.68	3290722	C10 H16 O	152.1196	152.1195	96.4	100	818	4695939	10
(+)-4-Carene	13.05	99994719	**C10 H16**	136.1247	136.1246	97.2	99.8	863	3.08E+08	10
(+)-4-Carene	15.03	37343743			136.1246	97.5	99.8	878	1.24E+08	10
(1R,7S,E)-7-Isopropyl-4,10-dimethylenecyclodec-5-enol	26.44	4465776	**C15 H24 O**	220.1822		93.5	99	695	970506	10
(1R,7S,E)-7-Isopropyl-4,10-dimethylenecyclodec-5-enol	26.66	8624626				95.4	99.5	777	278876	7.5
(1R,7S,E)-7-Isopropyl-4,10-dimethylenecyclodec-5-enol	26.81	6000373				93.3	96.9	727	205246	7.5
(2R,4R)-4-Methyl-2-(2-methylprop-1-en-1-yl)tetrahydro-2H-pyran	15.71	169812	C10 H18 O	154.1352	154.1352	91.8	90.4	783	590.5	10
(3R,6R)-3-Hydroperoxy-3-methyl-6-(prop-1-en-2-yl)cyclohex-1-ene	30.12	1163090	C10 H16 O2	168.1145		92.1	98.9	625	78973	10
(E)-1-(2,3,6-trimethylphenyl)buta-1,3-diene (TPB, 1)	22.82	661569	C13 H16	172.1247	172.1246	94.3	94.1	831	195305	10
(E)-1-(4-Hydroxy-3-methoxyphenyl)hexadec-3-en-5-one	24.39	809765	C23 H36 O3	360.2659		91	96	627	337828	7.5

1-(2,3-Dimethylphenyl)ethanone	19.07	1432233	C10 H12 O	148.0883	148.0886	95	99.2	767	1030816	10
1-(p-Toluidino)-1-deoxy-β-d-idopyranose	24.38	1849499	C13 H19 N O5	269.1258		93.1	98.8	679	20352	10
1, 1, 5-Trimethyl-1, 2-dihydronaphthalene	21.7	675419	C13 H16	172.1247	172.1246	96.8	97.1	899	203670	10
1,1-Diethyl-1,2,3,4-tetrahydronaphthalene	21.12	182684	C14 H20	188.156		94.8	100	738	590.5	10
1,2-Benzenediol, O-(4-ethylbenzoyl)-O'-propargyloxycarbonyl-	18.93	7207512	C19 H16 O5	324.0992		96	98.6	828	38885427	10
1,2-Benzenediol, O,O'-di(4-butylbenzoyl)-	25.83	316108	**C28 H30 O4**	430.2139		95.4	96	848	144996	7.5
1,2-Benzenediol, O,O'-di(4-butylbenzoyl)-	26.51	225044		430.2139		92.4	92.2	772	169464	5
1,2-Cyclohexanediol	10.31	156142	C6 H12 O2	116.0832		93.7	100	686	590.5	10
1,3-Cyclohexadiene, 5-butyl-	13.47	5670893	C10 H16	136.1247	136.1246	92.1	100	606	44491606	10
1,6-Octadiene, 8-methoxy-	15.42	12176554	C9 H16 O	140.1196		92	100	600	2466961	10
11,12-Dioxatetracyclo[4.3.1.1(3,10).1(2,5)]dodecane	22.33	2318509	C10 H14 O2	166.0988		91.1	97.3	609	73370	10
11-Oxatetracyclo[5.3.2.0(2,7).0(2,8)]dodecan-9-one	24.42	8965041	C11 H14 O2	178.0988	178.0988	91.9	98.8	618	179348	10
1-Adamantanecarboxylic acid, 3-phenyl-2-propenyl ester	17.69	8431296	C20 H24 O2	296.1771		92.7	99.9	637	15356941	10
1-Cyclohexene-1-carboxaldehyde, 2,6,6-trimethyl-	16.52	2643016	C10 H16 O	152.1196	152.1198	95.2	100	759	2291016	10
1H-1,2,3,4-Tetrazole-1-propanoic acid, 2-(4-fluorophenyl)-2-oxoethyl ester	24.31	336930	C12 H11 F N4 O3	278.081		97	97.5	899	129787	7.5
1H-Indene, 2,3-dihydro-1,1,5,6-tetramethyl-	17.27	1161807	**C13 H18**	174.1403	174.1403	94.5	95.2	817	7057297	10
1H-Indene, 2,3-dihydro-1,1,5,6-tetramethyl-	20.9	997626			174.1403	96	98.4	831	61564	10

1H-Indene, 2,3-dihydro-1,1,5,6-tetramethyl-	21.54	3493691			174.1403	96.9	99.3	859	311843	10
1-Octen-3-ol	11.97	28277298	C8 H16 O	128.1196		95.2	99.9	764	1.16E+08	10
1-Oxaspiro[4.5]dec-6-ene, 2,6,10,10-tetramethyl-	20.46	1204388	**C13 H22 O**	194.1665		94.6	100	728	590.5	10
1-Oxaspiro[4.5]dec-6-ene, 2,6,10,10-tetramethyl-	20.83	1405544				95	99.6	759	59301	10
1-Propene, 3,3'-oxybis-	7.79	197081	C6 H10 O	98.07262		93.6	99.6	686	822883	7.5
1-Tridecyn-4-ol	7.78	218216	C13 H24 O	196.1822		93	99.8	655	1061640	7.5
1-Trifluoroacetoxy-10-undecene	14.66	11040534	C13 H21 F3 O2	266.1488		90.1	94.8	607	2471885	10
2(4H)-Benzofuranone, 5,6,7,7a-tetrahydro-4,4,7a-trimethyl-	25.05	4289360	C11 H16 O2	180.1145	180.1144	97.2	99.9	862	1072251	7.5
2,3-Butanediol, [S-(R*,R*)]-	5.55	202927	**C4 H10 O2**	90.06753		98.1	99.8	906	590.5	2.5
2,3-Butanediol, [S-(R*,R*)]-	5.9	186871				99.1	100	953	176301	7.5
2,4,6-Cycloheptatrien-1-one, 2-hydroxy-	17.25	8410760	C7 H6 O2	122.0362	122.0362	96.1	99.8	810	44840878	10
2',4'-Dihydroxy-3'-methylbutyrophenone	22.91	184329	C11 H14 O3	194.0938	194.0937	91.7	95.1	683	590.5	10
2,4-Heptadienal, (E,E)-	12.47	1453138	C7 H10 O	110.0726	110.0727	94.7	99.8	738	1955883	10
2,5-Furandione, dihydro-3-methyl-	9.71	338810	C5 H6 O3	114.0312		95.1	100	753	590.5	7.5
2,5-Pyrrolidinedione, 1-[(3,4-dimethylbenzoyl)oxy]-	26.14	661359	C13 H13 N O4	247.0839		95.1	98.7	782	47468	10
2,6-Nonadienal, (E,Z)-	16.81	506196	C9 H14 O	138.1039		91.4	97.8	612	590.5	10
2,7-Nonadien-5-one, 4,6-dimethyl-	24.34	239889	C11 H18 O	166.1352		92.8	97	698	244656	10
2-Acetoxymesitylene	25.34	389589	C11 H14 O2	178.0988		93.1	100	656	138355	7.5
2-Buten-1-one, 1-(2,6,6-trimethyl-1,3-cyclohexadien-1-yl)-, (E)-	22.21	3813503	C13 H18 O	190.1352	190.1352	93.3	97	726	98107	5
2-Cyclohexen-1-ol, 3-methyl-6-(1-methylethyl)-	18.28	2569518	C10 H18 O	154.1352		94.9	99.9	747	814172	7.5
2H-1-Benzopyran, 3,4,4a,5,6,8a-hexahydro-	20.22	2544386851	**C13 H22 O**	194.1665	194.1667	97.1	99.9	858	17721606	10

2,5,5,8a-tetramethyl-, (2α,4aα,8aα)-									
2H-1-Benzopyran, 3,4,4a,5,6,8a-hexahydro-2,5,5,8a-tetramethyl-, (2α,4aα,8aα)-	21.1	22090284			194.1667	95.7	100	786	15412	10
2H-Pyran-2-one, 4,6-dimethyl-	17.7	7230368	C7 H8 O2	124.0519	124.0519	93.7	99.9	686	1032637	10
2'-Hydroxy-4',5'-dimethylacetophenone	16.81	217163	C10 H12 O2	164.0832	164.0832	94.3	99.6	722	21828	2.5
2-Methyl-3,5-dinitrophenyl β-phenylpropionate	22.97	514044	C16 H14 N2 O6	330.0846		92.8	98.4	670	126684	5
2-Methyl-5-(propan-2-ylidene)cyclohexane-1,4-diol	20.78	294723	C10 H18 O2	170.1301		92.3	99	636	1483519	10
2-Naphthalenol, 1,2,3,4,4a,5,6,7-octahydro-2,5,5-trimethyl-	22.75	54867978	C13 H22 O	194.1665		94.7	99.9	735	1390848	10
2-Oxatricyclo[3.3.1.1(3,7)] decane, 1-methyl-	16.78	219818	C10 H16 O	152.1196	152.1198	92.6	98.5	661	268896	7.5
2-Oxepanone, 4-methyl-	14.64	15760355	C7 H12 O2	128.0832		94.2	99.4	722	219116	7.5
2-Pentene, 1-ethoxy-4-methyl-, (Z)-	14.9	1698430	C8 H16 O	128.1196		94.9	99.8	746	1768899	10
2-Pyrazoline-3-carboxylic acid, 5-hydroxy-1-(4-methylbenzoyl)-5-phenyl-, methyl ester	24.67	10110454	C19 H18 N2 O4	338.1261		92.4	100	619	5065833	10
3(10)-Caren-4-ol, acetoacetic acid ester	22.04	13041833	C14 H20 O3	236.1407		92.6	99.7	635	14509902	10
3-(2-Isopropyl-5-methylphenyl)-2-methylpropionic acid	22.36	1172278	**C14 H20 O2**	220.1458		91.5	96.7	638	98722	7.5
3-(2-Isopropyl-5-methylphenyl)-2-methylpropionic acid	25.95	2886280				92.6	98.7	658	1029137	10
3-(tert-Butyl)-4-methoxyphenyl acetate	21.53	3818242	C13 H18 O3	222.1251		91.8	98.7	616	590.5	7.5
3-Octanone	12.1	20400043	C8 H16 O	128.1196		95.7	100	782	5323114	10

3-tert-Butyl-4-hydroxyanisole, acetate	24.22	935657	C13 H18 O3	222.1251		94.5	98.4	758	4650742	10
4-Acetoxy-3-methoxyacetophenone	24.29	2608498	C11 H12 O4	208.073		93.6	94.8	783	24669935	10
4-Ethylbenzoic acid, tridec-2-ynyl ester	24.33	166793	C22 H32 O2	328.2397		95.6	100	777	496077	7.5
4-Hydroxy-2,4,5-trimethyl-2,5-cyclohexadien-1-one	19.71	576946	C9 H12 O2	152.0832	152.0832	94.2	99.8	711	330904	7.5
4-Hydroxy-2,6,6-trimethyl-3-oxocyclohexa-1,4-dienecarbaldehyde	19.85	6856667	**C10 H12 O3**	180.0781	180.078	92.4	99.9	621	82433	2.5
4-Hydroxy-2,6,6-trimethyl-3-oxocyclohexa-1,4-dienecarbaldehyde	21.88	65163900			180.0781	94.9	99.9	745	98932	10
4-Isopropyl-5,5-dimethyl-5H-furan-2-one	24.17	1839516	C9 H14 O2	154.0988		92.7	100	635	1035989	10
4-Methylbenzoic acid, 3-pentyl ester	17.21	352132	C13 H18 O2	206.1301		92.3	100	615	2218367	10
5,7-Dimethyl-1,3-adamantanediol	20.83	242085	C12 H20 O2	196.1458		92.2	98.5	640	35805	7.5
5-Acetyl-4,6,6-trimethylcyclohexa-2,4-dienone	25.65	1916312	C11 H14 O2	178.0988	178.0988	92.9	99.8	645	12730	7.5
5-Hepten-2-ol, 6-methyl-	12.37	3204024	C8 H16 O	128.1196		95.3	99.8	769	485881	10
5-Isopropyl-2-methylbicyclo[3.1.0]hexan-2-ol #	17.56	653007901	C10 H18 O	154.1352	154.1353	96.1	100	802	3.52E+08	10
6,10,14-Trimethyl-2-pentadecanol, TMS derivative	18.99	1476927	C21 H46 O Si	342.3312		90.4	92.4	671	328878	10
6,8-Nonadien-2-one, 6-methyl-5-(1-methylethylidene)-	22.98	684681	**C13 H20 O**	192.1509	192.1508	93.2	99.5	669	590.5	10
6,8-Nonadien-2-one, 6-methyl-5-(1-methylethylidene)-	23.3	27732850			192.1508	94.2	98.5	741	42480	10
6-Hydroxy-4-methylcoumarin	24.46	699063	C10 H8 O3	176.0468	176.0467	96.2	99.2	826	132428	7.5

7-Methylenebicyclo[4.2.0] octane	21.49	950929	C9 H14	122.109	122.1092	94.1	97.4	758	91110	7.5
8,9-Dehydrothymol methyl ether	19.08	360941	C11 H14 O	162.1039	162.1039	95.4	98.1	805	588960	7.5
8a-Methyl-5-methylene-3-([(pyridin-2-ylmethyl)-amino]-methyl)-decahydro-naphtho[2,3-b]furan-2-one	13.48	11803671	C21 H28 N2 O2	340.2145		92.4	100	619	47663839	10
Alanine, N-methyl-N-ethoxycarbonyl-, isobutyl ester	27.65	647625	C11 H21 N O4	231.1465		95.8	98.6	815	49205	7.5
Ascaridole	19.02	1200727	C10 H16 O2	168.1145		92.9	98.8	667	929122	10
Aspirin methyl ester	17.83	1923812	C10 H10 O4	194.0574		96.6	98.9	852	2660526	10
Benzene, 1-(1-formylethyl)-4-(1-buten-3-yl)-	19.52	897711	C13 H16 O	188.1196		94.8	99	758	85489	10
Benzene, 1-ethenyl-4-ethyl-	15.23	11737598	C10 H12	132.0934	132.0934	97.2	99.7	862	47715340	10
Benzene, 1-methyl-3-(1-methylethenyl)-	15.14	17661026	C10 H12	132.0934	132.0934	98	99.8	901	49655548	10
Benzene, 2-methoxy-4-methyl-1-(1-methylethyl)-	18.74	11495142	C11 H16 O	164.1196	164.1195	97.5	99.9	877	590.5	10
Benzene, 2-methoxy-4-methyl-1-(1-methylethyl)-	18.96	58346104	C11 H16 O	164.1196	164.1195	97.5	100	876	9724510	10
Benzeneacetic acid, 2-butyl ester	11.08	1540939	C12 H16 O2	192.1145		92.9	100	643	590.5	7.5
Benzofuran, 2,3-dihydro-	18.48	47205292	C8 H8 O	120.057	120.0569	96.8	99.9	843	2361969	10
Benzoic acid, 2-formyl-, methyl ester	21.9	22529268	C9 H8 O3	164.0468	164.0467	94.6	99	751	482158	10
Benzoic acid, 4-ethoxy-, ethyl ester	24.93	619083	C11 H14 O3	194.0938	194.0937	90.2	87.2	768	1420400	10
Benzoic anhydride, 4,4',6,6'-tetramethoxy-2,2'-dimethyl-	24.48	143140	C20 H22 O7	374.136		94.4	99.5	729	590.5	2.5
Benzyl alcohol	13.58	181223	C7 H8 O	108.057	108.0569	97.9	98.8	917	954217	7.5
Bicyclo[2.2.1]heptan-2-ol, 1,7,7-trimethyl-, acetate, (1S-endo)-	20.1	82209313	C12 H20 O2	196.1458		97	100	849	175009	10

Bicyclo[3.1.0]hex-2-ene, 2-methyl-5-(1-methylethyl)-	10.2	14288491	**C10 H16**	136.1247	136.1246	96.4	99.6	826	98709608	10
Bicyclo[3.1.0]hex-2-ene, 2-methyl-5-(1-methylethyl)-	12.72	26183480			136.1246	93	92.9	791	87921904	10
Bicyclo[3.1.0]hexan-2-ol, 2-methyl-5-(1-methylethyl)-, (1α,2α,5α)-	14.6	109760868	C10 H18 O	154.1352	154.1352	96.9	100	843	1.5E+08	10
Bicyclo[3.1.0]hexane, 4-methylene-1-(1-methylethyl)-	11.7	8150588	C10 H16	136.1247	136.1246	96.7	99.1	851	661578	10
Bicyclo[3.1.1]hept-2-en-6-ol, 2,7,7-trimethyl-, acetate, [1S-(1α,5α,6β)]-	17.12	480969	C12 H18 O2	194.1301		92.8	100	637	33502	2.5
Bicyclo[3.1.1]heptan-2-one, 3,6,6-trimethyl-	17.97	2913251	C10 H16 O	152.1196	152.1195	93.6	100	678	4120972	10
Butanoic acid, 3-(1-phenylethoxy)-	24.4	737003	C12 H16 O3	208.1094		95.7	98.8	811	590.5	10
Carvenone	19.47	4296281	C10 H16 O	152.1196	152.1195	95.2	100	761	9041219	10
Carvone	19.11	163620393	C10 H14 O	150.1039	150.1039	93.4	95.3	762	1.82E+08	10
Cinnamyl cinnamate	17.29	11870718	C18 H16 O2	264.1145		92.1	99.8	611	3571019	10
Coumarin-6-carboxaldehyde	27.38	3227758	C10 H6 O3	174.0312	174.0309	93.1	89.9	855	442516	10
Cyclohexan-1-ethanol, 1-hydroxymethyl-	15.91	258498	C9 H18 O2	158.1301		90.1	94	626	89792	5
Cyclohexanecarboxylic acid, 2-methylphenyl ester	17.96	253463	C14 H18 O2	218.1301		91.4	90.6	755	1609117	10
Cyclohexaneethanol, 2-methylene-	18.28	2203009	C9 H16 O	140.1196		93.5	99	697	590.5	10
Cyclohexanol, 1-methyl-4-(1-methylethylidene)-, acetate	16.56	2421205	C12 H20 O2	196.1458		92.6	99.8	635	90809	5
Cyclohexanol, 5-methyl-2-(1-methylethyl)-, (1α,2β,5α)-(±)-	17.47	190455993	C10 H20 O	156.1509		91.8	93	729	3.38E+08	10
Cyclopentaneacetic acid, 3-oxo-2-pentyl-, methyl ester	26.5	988472	C13 H22 O3	226.1564		91.6	98.4	613	1664291	10
endo-Borneol	17.34	665342180	C10 H18 O	154.1352		96.9	100	843	3.64E+09	10
Eucalyptol	13.49	137379678	C10 H18 O	154.1352	154.1353	96.1	99.8	806	81074268	10

Eugenol	21.61	15666829	C10 H12 O2	164.0832	164.0832	97.8	99.9	890	357378	10
Furan, 2-hexyl-	12.91	1674703	C10 H16 O	152.1196		94.8	99.9	742	530786	10
Isobutyl 3-(perhydro-5-oxo-2-furyl)propionate	20.9	1499440	C11 H18 O4	214.12		91.7	96.7	651	61564	10
Isophthalic acid, ethyl 2-isopropoxyphenyl ester	24.25	2400810	C19 H20 O5	328.1305		96.2	100	807	2465209	10
L-(-)-Fucose, tetrakis(trifluoroacetate), benzyloxime (isomer 1)	16.55	4000563	C21 H15 F12 N O9	653.055		92.7	99.4	648	963742	10
Levoglucosenone	15.69	694370	C6 H6 O3	126.0312		94.3	99.5	724	207804	10
Linalool	15.41	68180082	C10 H18 O	154.1352		96.4	100	818	28282051	10
Methyl isovalerate	5.14	386385	C6 H12 O2	116.0832		96	100	801	92486	5
Methyl nicotinate	16.47	2140331	C7 H7 N O2	137.0471	137.0472	96.3	100	812	156879	10
Mexacarbate	21.59	1593504	C12 H18 N2 O2	222.1363		92.7	100	635	98421	10
Myristic acid, 4-methoxyphenyl ester	24.85	441132	C21 H34 O3	334.2503		92.7	100	635	1690115	10
Naphthalene, 1,2,3,4-tetrahydro-1,1,6-trimethyl-	19.38	2090448	**C13 H18**	174.1403	174.1403	95.9	99.4	807	66268	10
Naphthalene, 1,2,3,4-tetrahydro-1,1,6-trimethyl-	20.3	337900			174.1403	94.4	95	820	590.5	7.5
Naphthalene, 1,2,3,4-tetrahydro-1,6-dimethyl-4-(1-methylethyl)-, (1S-cis)-	26.55	222037	C15 H22	202.1716	202.1716	96.8	100	837	590.5	10
Nonanoic acid	19.93	1557642	C9 H18 O2	158.1301		94.2	99.9	712	82433	2.5
o-Cymene	13.27	164881269	**C10 H14**	134.109	134.109	97.5	99.8	876	64395057	10
o-Cymene	22.48	189534			134.1093	97.7	99	903	590.5	10
Oxalic acid, diallyl ester	20.84	225581	C8 H10 O4	170.0574		91.3	96.7	631	590.5	7.5
Oxirane, 2-(hexyn-1-yl)-3-methoxymethylene-	16.66	362141	C10 H14 O2	166.0988		91.4	98.4	600	471839	7.5
p-Cymene-2,5-diol	25.35	11687997	C10 H14 O2	166.0988	166.0989	94.4	99.7	726	32562662	10
Pentafluoropropionic acid, nonyl ester	17.33	27888558	C12 H19 F5 O2	290.13		92.1	98.1	641	59842059	10
Phenacylidene diacetate	11.3	11381000	C12 H12 O5	236.0679		97.1	100	856	2867571	10
Phenol, 2-methyl-5-(1-methylethyl)-	20	30425359	**C10 H14 O**	150.1039	150.1039	96.2	99.9	810	5615221	10
Phenol, 2-methyl-5-(1-methylethyl)-	20.41	333047398			150.1039	96.8	99.8	841	5.18E+08	10
Phenol, 5-ethenyl-2-methoxy-	20.65	31455201	C9 H10 O2	150.0675	150.0675	97.3	99.6	873	558170	10
Phenylethyl Alcohol	15.74	6830645	C8 H10 O	122.0726	122.0727	96.6	98.6	859	2254506	10

Phenylglyoxal	11.38	21808482	C8 H6 O2	134.0362		96.8	99.4	853	2867571	10
p-Mentha-1,5,8-triene	15.77	5306778	C10 H14	134.109	134.109	94.6	99.1	748	2254506	10
p-Mentha-1,5,8-triene	16.36	2034565	C10 H14	134.109	134.109	95	98	792	4235996	10
Propanedioic acid, nitrile, hydrazide, N2-(1-oxo-3-phenyl-2-propenyl)-	17.6	4853291	C12 H11 N3 O2	229.0846		95.5	99.9	776	7874932	10
Propanoic acid, 2-methyl-, 3-hydroxy-2,2,4-trimethylpentyl ester	21.52	2925114	**C12 H24 O3**	216.172		91.9	97.5	645	1072010	7.5
Propanoic acid, 2-methyl-, 3-hydroxy-2,2,4-trimethylpentyl ester	22.04	5015401				93.9	99.3	709	14509902	10
Proximpham	14.93	940745	C10 H12 N2 O2	192.0893		96.5	100	826	6439903	10
p-Sec-butylphenyl acetate	20.86	444407	C12 H16 O2	192.1145		94	99.5	708	309494	10
p-Toluic acid, tridec-2-ynyl ester	19.05	2610587	C21 H30 O2	314.224		94.4	100	719	1503936	10
Pyridine, 3-ethenyl-	11.76	1007170	C7 H7 N	105.0573	105.0573	95.4	99	788	189182	10
Quinoline, 3-methyl-	27.67	740915	C10 H9 N	143.073	143.0729	97.8	99.1	908	74972	10
Spiro[2.4]heptane, 1,5-dimethyl-6-methylene-	10.94	1554717	C10 H16	136.1247		94.6	99.8	734	433309	10
Terbucarb	23.87	4961974	C17 H27 N O2	277.2036		91.7	98.7	611	9401780	10
Thymol	20.23	6039299922	C10 H14 O	150.1039	150.1039	96.3	99.8	819	8.41E+09	10
Thymoquinone	19.22	440052463	C10 H12 O2	164.0832	164.0832	96.1	99.3	819	5.97E+08	10
trans-Sinapyl alcohol	29.41	236641	C11 H14 O4	210.0887	210.0886	93.5	98.6	701	9914	5
Tributyl phosphate	24.71	26822259	C12 H27 O4 P	266.1642		93.3	99.5	676	962299	7.5
Tricyclo[6.3.0.0(1,5)]undecan-10-one, 4-hydroxy-5,9-dimethyl-	27.09	12706073	C13 H20 O2	208.1458	208.1458	93.4	99.6	676	654252	10
Urea, (phenylmethoxy)-	13.45	11699731	**C8 H10 N2 O2**	166.0737		93.6	100	678	20258596	10
Urea, (phenylmethoxy)-	18.31	388417				96.7	100	836	457348	7.5
α-Calacorene	25.25	1214482	C15 H20	200.156	200.156	93.8	95.3	781	1583302	10
α-TERPINYL FORMATE	17.92	61399044	C11 H18 O2	182.1301		96.8	100	841	1.36E+08	10
β-Myrcene	12.23	1041641	C10 H16	136.1247		91.9	96.9	659	682492	7.5
γ-Terpinene	14.26	156676375	C10 H16	136.1247	136.1248	97.5	99.7	879	4.81E+08	10

Text S1. Additional characterization for TiO_2 MPs/SM, $CaCO_3$ MPs/SM and pristine particles (i.e. TiO_2 and $CaCO_3$ without SM).

TiO_2 MPs/SM. The XRD pattern of TiO_2 MPs/SM powder, recorded using Mo Kα radiation, shows broad peaks centred at 11.74° (25.67° Cu Kα), 17.45° (38.47°), 21.77° (48.43°), 24.60° (55.12°), 27.91° (63.17°), 30.54° (69.78°), 32.90° (75.91°), and 35.63° (83.29°). All the detected reflections can be attributed to the anatase phase of TiO_2, with the most intense peak at 11.74° (25.67° Cu Kα), corresponding to the (101) plane (ICDD Card No. 01-075-2552) [1–3]. The remaining signals were assigned to (112), (200), (211), (204), (220), (301), and (312) planes. Nevertheless, a minor presence of brookite (~10%) cannot be entirely ruled out, since the peaks at 11.74° and 21.77° may also correspond to the (111) and (231) planes of this TiO_2 polymorph (ICDD Card No. 04-022-2622) [3,4]. However, the absence of the typical high-intensity doublet characteristic of the brookite phase suggests that the sample is predominantly composed of the anatase form (~90%).

Raman spectroscopy provided further confirmation of the TiO_2 crystalline phases (Figure 2B and Table 1 in the text). The spectrum revealed the presence of characteristic bands of the anatase polymorph, confirming the results obtained by XRD analysis. A strong band was observed at 146 cm^{-1}, corresponding to the E_g vibrational mode of anatase, along with the B_{1g} mode at 411 cm^{-1}. Additional bands were detected at 517 cm^{-1} and 623 cm^{-1}, attributable to the A_{1g} + B_{1g} and E_g modes of the anatase phase, respectively [5–7]. Two minor shoulders were also observed between 219 cm^{-1} and 279 cm^{-1}, which can be attributed to the B_{1g} and B_{2g} vibrational modes of brookite, suggesting the possible presence of this secondary polymorph in low concentration [7,8]. Nevertheless, a partial contribution from the low-frequency E_g mode of anatase, typically appearing as a broad shoulder around 200 cm^{-1}, cannot be excluded [5–7]. In addition, the Raman spectrum exhibited broad fluorescence related features at~874,~1627, and~3352 cm^{-1}, which are likely associated with the vibrational modes of the organic functional groups/phase controllers used to functionalize/draw the particles (see **Figure S1** in Supplementary information). Specifically, the band at 874 cm^{-1} may correspond to ν(C–C), ν(C–O) stretching and δ(C–H) bending vibrations, while the band at 1627 cm^{-1} may arise from ν(C=C) or ν(C=O) stretching, as well as δ(O–H) bending [9–18]. The broad signal at 3352 cm^{-1} is attributable to ν(O–H) stretching vibrations, indicative of hydroxyl groups of phytocompounds based activators [19]. FTIR-ATR analysis confirmed the successful synthesis and functionalization of TiO_2 microparticles with *Satureja montana* extract. A broad and intense absorption band observed in the range 800–600 cm^{-1} was attributed to Ti–O–Ti stretching and bending vibrations, typical of titanium dioxide frameworks [20,21]. Signals at approximately 3400 cm^{-1} and 1630 cm^{-1} were assigned to O–H stretching and bending vibrations, respectively, indicating the presence of hydroxyl groups, either due to surface hydroxylation or originating from phenolic and terpenoid constituents of the plant extract [22–26]. Additionally, the spectrum displayed several distinctive bands associated with phytocompounds from the extract. Notably, peaks at 2974 cm^{-1} and 2893 cm^{-1} correspond to the asymmetric stretching of $–CH_3$ groups and the symmetric stretching of $–CH_2$ groups, respectively. Absorption bands between 1454 and 1395 cm^{-1} were assigned to δ(C–H) bending vibrations, specifically the asymmetric and symmetric bending modes of methyl and methylene groups. Furthermore, the band at 1268 cm^{-1} was attributed to ν(C–O) stretching vibrations, while the signal at 1051 cm^{-1} indicated ν(C–O) or ν(C–C) stretching modes [25,26].

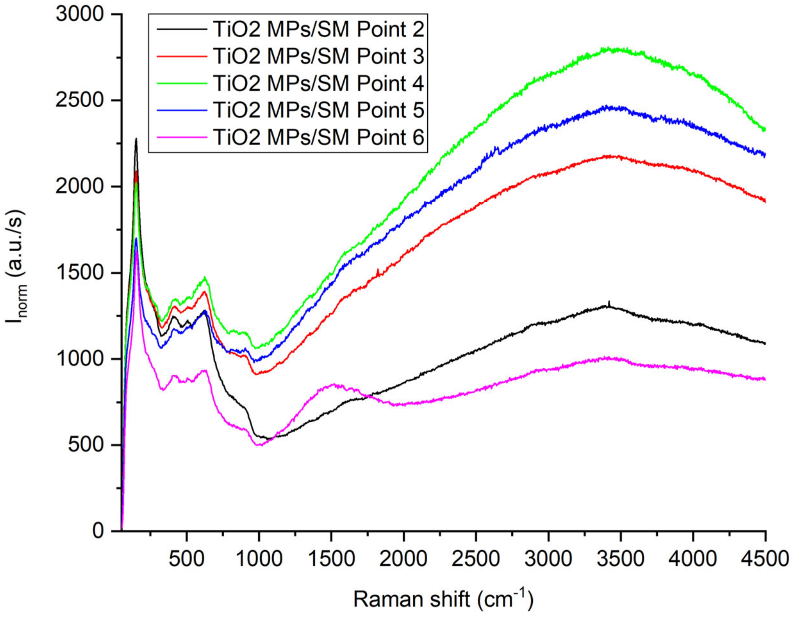

Figure S1. Raman spectrum of the organic activators/phase controllers' fingerprint into TiO_2 MPs/SM sample (where Anatase is the major component, as reported in the full text). Raman spectra were collected at different points of the TiO_2 MPs/SM sample. The spectra were normalized based on the integration time used (120 s for points 2 and 3, and 60 s for points 4, 5, and 6).

The presence of organic matter induces a strong fluorescence background in the spectra collected at various points (**Figure S1**), which limits the identification of functional groups by Raman spectroscopy. These bands are more effectively characterised using complementary techniques such as FTIR.

Figure S2 also reports the SEM/EDX microanalysis characterization of the TiO_2 MPs/SM sample.

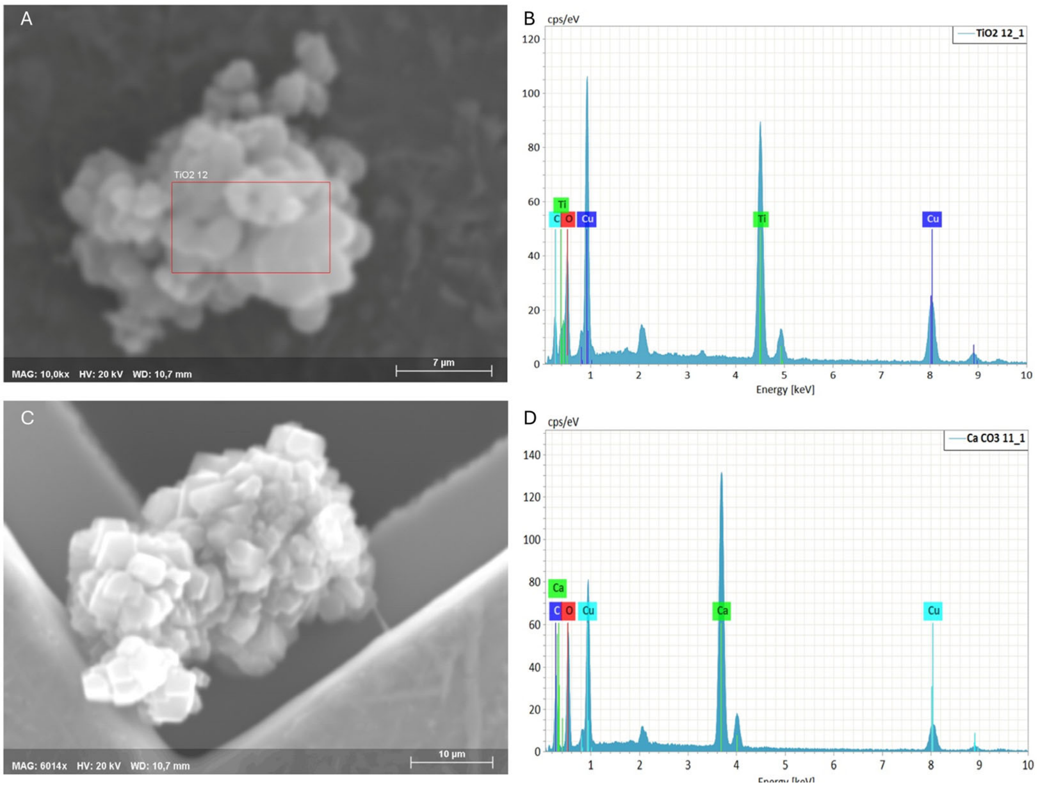

Figure S2. TiO_2 MPs/SM: (**A**) micrograph and (**B**) EDX analysis. $CaCO_3$ MPs/SM: (**C**) micrograph and (**D**) EDX analysis for calcite.

$CaCO_3$ MPs/SM. The XRD diffractogram obtained for $CaCO_3$ MPs/SM reveals calcite as the predominant crystalline phase. The most intense diffraction peak is observed at 2θ = 29.43°, corresponding to the (104) plane, a characteristic reflection of calcite (ICCD Card No: 00–005-0586) [27–30]. Additional peaks, including those at 31.46°, 56.54°, and beyond 58.20°, exhibit lower intensities. Nevertheless, the overall diffraction pattern unambiguously confirms the presence of calcite, with no significant traces of other $CaCO_3$ polymorphs [27–30]. These findings suggest that *Satureja montana* extract favors the formation of the calcitic phase over other crystalline structures (i.e. predominant phase). Complementary insights were provided by Raman spectroscopy, which revealed vibrational features attributable to both the calcitic phase and organic phytocompounds introduced by SM extract during synthesis. The presence of calcite is confirmed by the bands at 248 cm^{-1}, 718 cm^{-1}, and 1092 cm^{-1}. Specifically, the 1092 cm^{-1} band corresponds to the symmetric stretching vibration of the CO_3^{2-} group (A_{1g} mode), while the 718 cm^{-1} band is attributed to the in-plane bending vibration of the carbonate ion (E_g mode) [31,32]. The signal at 248 cm^{-1} relates to lattice translational vibrations (E_g mode) but may also partially overlap with skeletal torsional and deformation modes of aromatic phytocompounds, suggesting nanoparticle surface functionalization [9–13,17]. Moreover, other spectral bands support the presence of flavonoids and phenolic terpenoids on the carbonate surface. In the low-frequency region, several additional Raman bands (320, 412, 507, and 626 cm^{-1}) are assigned to skeletal deformations, twisting, and out-of-plane vibrations of aromatic ring systems [9–13,17]. The band at 746 cm^{-1} arises from a combination of ν(C–C) stretching, δ(C–O) bending, and aromatic ring deformation, while the signal at 890 cm^{-1} is linked to ν(C–OH) and ν(C–C) stretching, as well as δ(–CH) and δ(–CH_2) bending vibrations [9–13,17]. Other bands such as 1009 cm^{-1} and 1180 cm^{-1} are attributed to in-plane δ(–CH) bending, ν(C–O) stretching, and aromatic skeletal vibrations, whereas the band at 1339 cm^{-1} is associated with δ(–CH_3) bending (notably of isopropyl groups) and δ(–CH_2) scissoring modes. The FTIR spectrum further confirms the dual nature of the material, revealing contributions from both the inorganic carbonate phase and the organic constituents (Figure 4C, and Table 2). The bands at 1404 cm^{-1} and 870 cm^{-1} correspond to the asymmetric stretching and out-of-plane bending vibrations of CO_3^{2-}, respectively. The additional signal at 705 cm^{-1}, attributed to in-plane bending of CO_3^{2-}, confirms the identification of the calcite phase [33,34]. A broad band in the 3600–3000 cm^{-1} range is assigned to ν(O–H) stretching vibrations, originating from adsorbed water, surface hydroxyl groups, and phenolic or alcoholic phytocompounds. The presence of aliphatic chains, typical of plant-derived molecules, is confirmed by the bands at 2951, 2922, and 2856 cm^{-1}, which are associated with asymmetric and symmetric stretching vibrations of methyl –CH_3 and methylene –CH_2 groups [22–26]. A sharp absorption band at 1710 cm^{-1} corresponds to carbonyl ν(C=O) stretching, while the band at 1637 cm^{-1} is mainly due to molecular water bending δ(O–H), with potential contributions from ν(C=O) and ν(C=C) stretching vibrations in aromatic systems [22–26]. Finally, the bands at 1231 cm^{-1} and 1070 cm^{-1} are assigned to ν(C–O) and ν(C–C) stretching modes, as well as to skeletal vibrations of aromatic rings, further confirming the presence of oxygenated phytocompounds on the nanoparticle surface.

Similarly, to what was reported above for TiO_2 MPs/SM, also for the $CaCO_3$ MPs/SM microparticles the EDX characterization in microanalysis was carried out and reported in **Figure S2** (C and D).

Below, a detailed characterization of the brookite/anatase heterphase is also reported, where brookite represents the major component. This brookite/anatase heterophase was produced working at lower temperature (40 °C, according to **Scheme S1**) instead of 60°C, this latter applied during the anatase green synthesis route (reported in the manuscript). XRD analysis revealed a diffractogram (**Figure S3**) characterized by high background noise and broad peaks at 25.26°, 25.93°, 28.53°, and 47.11°. In particular, the peak at 28.69° may indicate the presence of brookite, as it lies close to the typical (121) reflection of this polymorph (30.8°). Peaks at 25.26°, 25.93°, and 47.11° were assigned to the (120), (111), and (231) planes, respectively [3,4]. However, the detection of the 28.69° reflection does not exclude the potential coexistence of anatase, whose most intense peak is commonly observed at 25.28° (101 plane), accompanied by a secondary reflection at 48.04° (200 plane) [1-3]. This sample was obtained under the same conditions as the anatase sample (presented in the text, see the corresponding XRD spectrum on Figure 2), but applying lower temperature (40°C instead of 60°C as reported in the manuscript in Materials and Methods section for TiO_2 MPs/SM enriched in anatase phase). In this working environment, a heterophase is formed, which is more abundant in brookite (~80%), rather than in anatase (~20%).

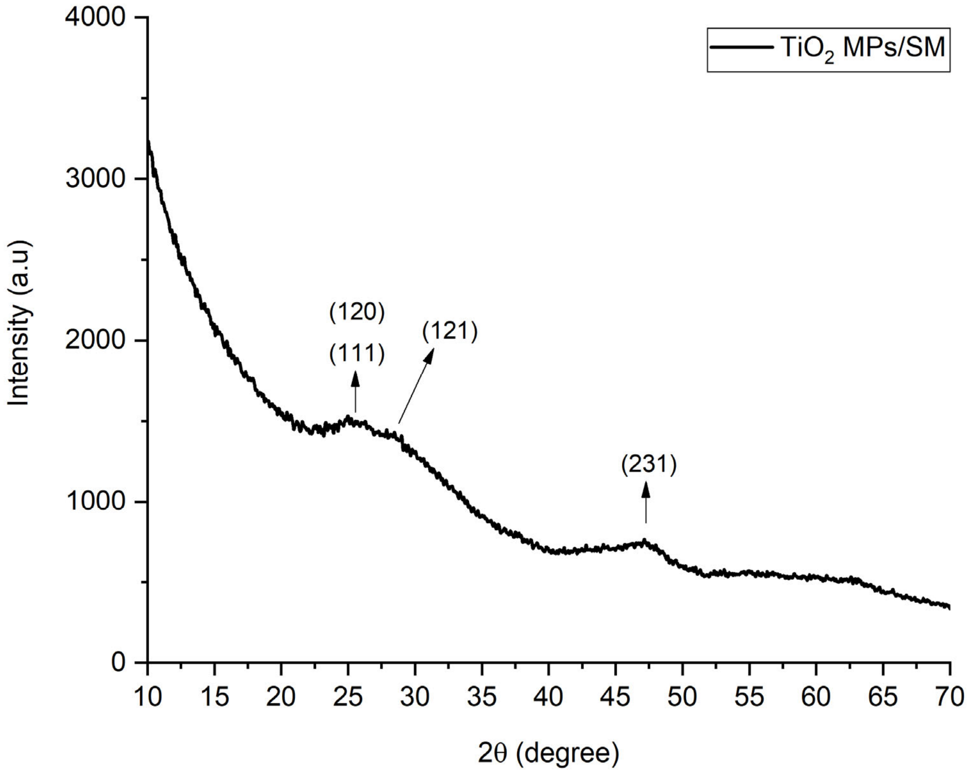

Figure S3. XRD of TiO_2 MPs/SM as brookite polymorphic crystalline form (i.e., mixed phase brookite/anatase, having brookite as the major component).

Raman spectroscopy provided further confirmation of the TiO_2 crystalline phases (**Figure S4** and **Table S2**). The spectrum featured characteristic brookite bands, including the A_{1g} mode at 224 cm^{-1} and B_{3g} modes at 287 and 312 cm^{-1}. Additional B_{2g} modes were observed at 386, 478, and 589 cm^{-1} [5,6]. A broad feature around 520 cm^{-1} and a weak signal at 643 cm^{-1} suggested the partial presence of anatase, whose E_g and A_{1g} modes typically appear around 516–520 cm^{-1} [6,35-37]. All detected bands were consistent with the

vibrational modes of the O–Ti–O network, including symmetric stretching (E_g), bending (B_{1g}, A_{1g}), wagging (B_{2g}), and twisting (B_{3g}) modes [5,6,36,37].

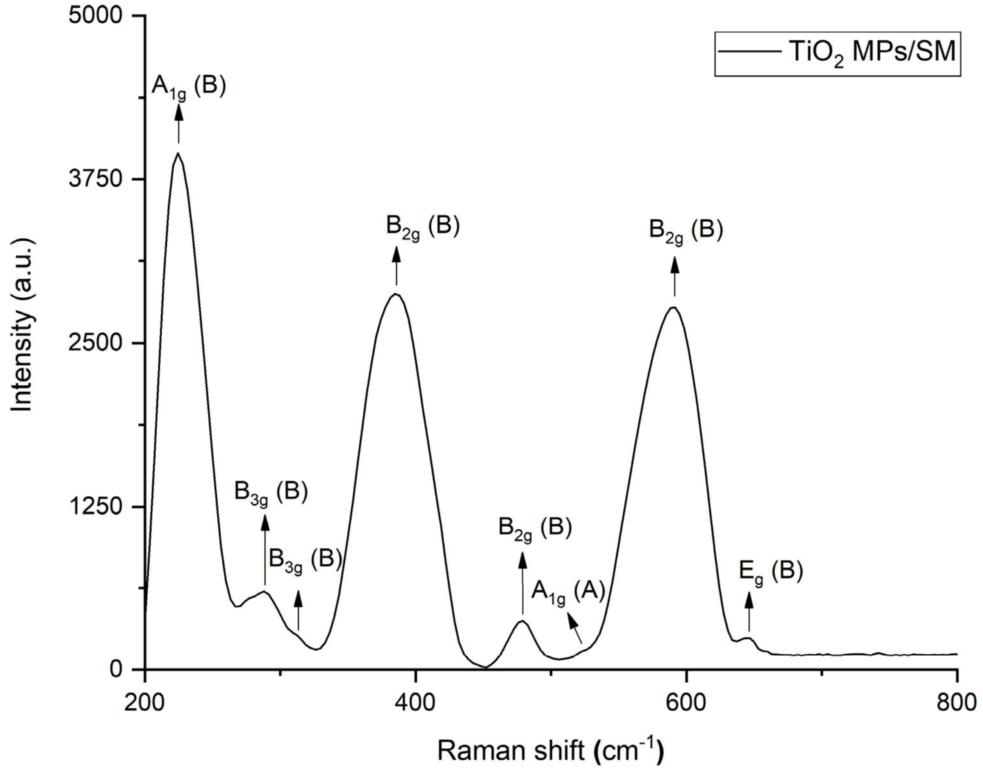

Figure S4. Raman spectrum of TiO_2 MPs/SM as heterogeneous phase brookite/anatase, having brookite as the major component.

Table S3. Raman band assignments of TiO_2 MPs /SM as heterogeneous phase brookite/anatase.

Band (cm^{-1})	Assignment	Ref.
224	A_{1g} mode (brookite)	[7,8]
287	B_{3g} mode (brookite)	[7,8]
312	B_{3g} mode (brookite)	[7,8]
386	B_{2g} mode (brookite)	[7,8]
478	B_{2g} mode (brookite)	[7,8]
520	E_g mode (anatase)	[8-10]
589	B_{2g} mode (brookite)	[7,8]
643	A_{1g} mode (anatase)	[8-10]

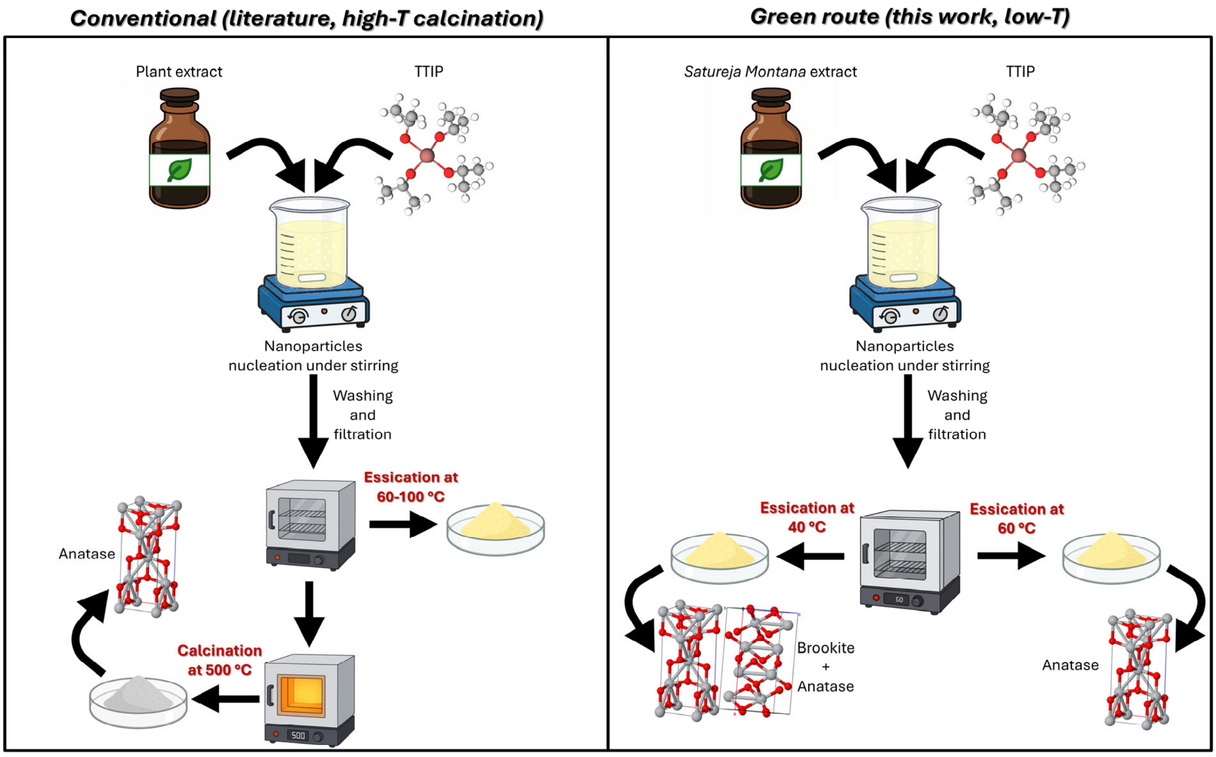

Scheme S1. Comparison between conventional high-temperature calcination synthesis and the green route developed in this work for TiO_2 production.

We also wanted to synthesise brookite/anatase heterophase, since many literatures works report an improved photocatalytic and antibacterial activity in the presence of a mixed brookite/anatase phase [36]. These heterophase results show good photocatalytic activity, as also reported in [36].

Finally, in this section we report the morphological characterization (see **Figure S5**) of the pristine particles (i.e., TiO_2 and $CaCO_3$ without SM, whose synthesis protocol is shown in **Scheme S2**).

The untreated (pristine) nanoparticles were synthesized via conventional precipitation methods in the absence of any plant extract or additional shape-directing agents. Their morphological features are presented in **Figure S5**. BET analysis (**Table 6** in the full text) confirms that these particles possess smaller dimensions and greater porosity compared to the extract-mediated ones. TiO_2 anatase particles retained a spherical shape in both cases, whereas $CaCO_3$ calcite particles showed more rounded edges and structural defects in the pristine form, in contrast to the sharper, more angular morphology observed when synthesized in the presence of the extract. This supports the role of the extract as a shape-directing agent, promoting the formation of particles with more defined edges and surface features.

While smaller size and higher porosity are typically associated with increased antimicrobial activity [38], this trend was not observed in our system (**Table 4**). Instead, the extract-mediated particles exhibited enhanced antimicrobial performance, which we attribute to the presence of sharper edges and surface defects, likely chemically activated by acidic functional groups such as carboxylic acids (precursors of ROS). These functional groups were absent in the pristine particles, as confirmed by acid–base titration. TGA and ζ-potential analyses also yielded no significant indications of surface activation, justifying their omission from **Table 5** in the full text.

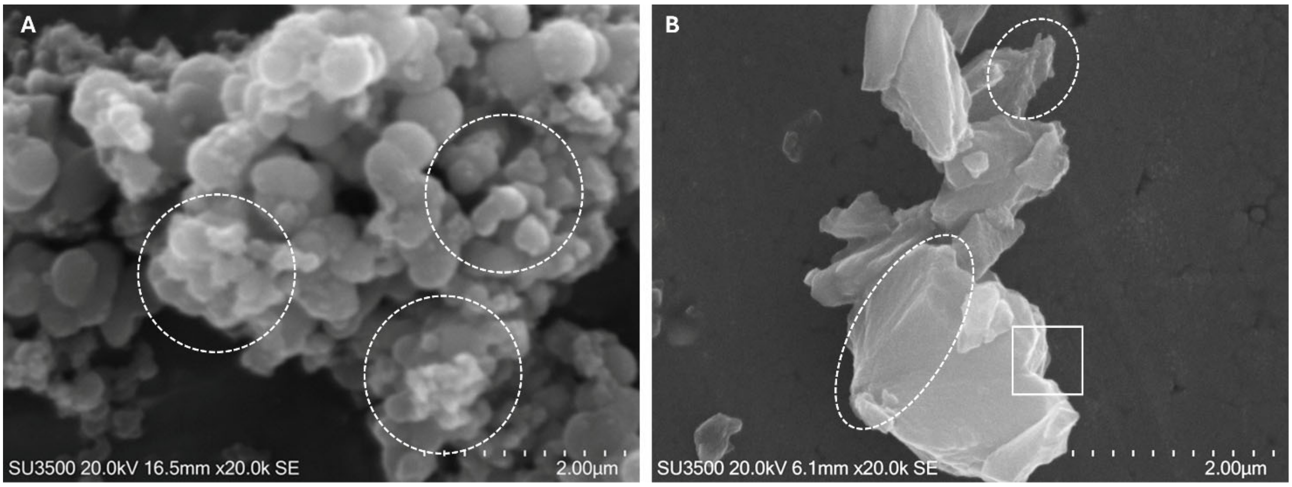

Figure S5. SE-micrographs of pristine particles. (**A**) Pristine TiO_2 Anatase particles cluster is shown. Dotted circles enclose particles with a diameter of about 200nm, magnification 20K. Bar 2 µm. (**B**) Pristine $CaCO_3$ Calcite particles present defects (square) and the edges are rounded and not sharp (dotted ovals), having dimensions of about 800nm, magnification 20K. Bar 2 µm.

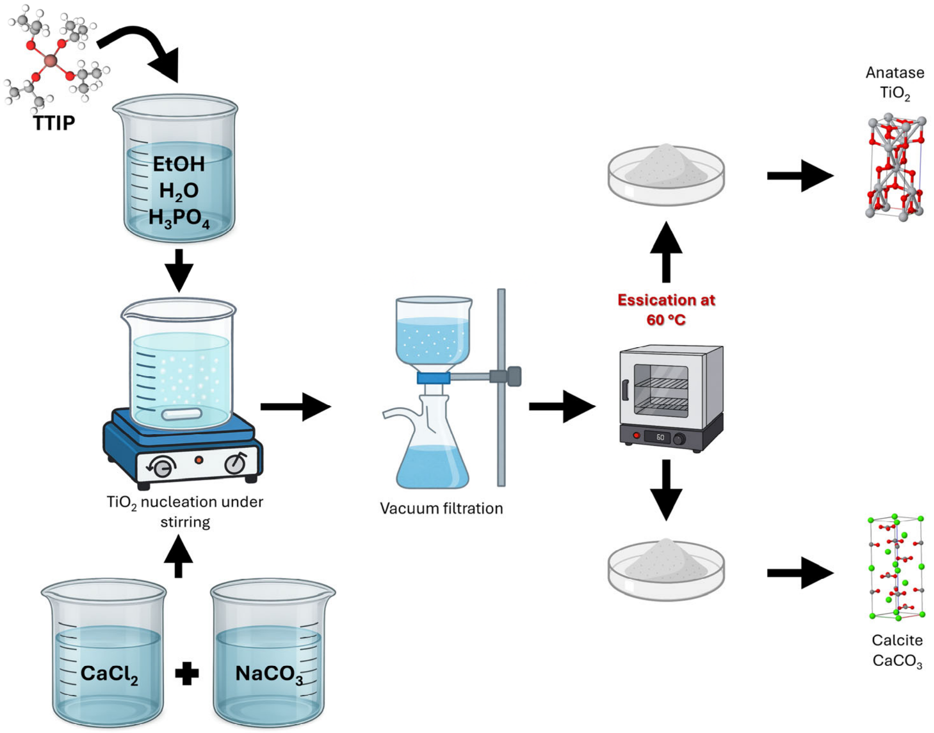

Scheme S2. Synthesis routes for pristine TiO_2 and $CaCO_3$. Top: anatase TiO_2 obtained by sol–gel hydrolysis of titanium(IV) isopropoxide (TTIP); bottom: calcite $CaCO_3$ produced by precipitation from $CaCl_2$ and $NaCO_3$.

Our results undoubtedly add important information that completes the state of the art, as for the first time, an aqueous extract of *Satureja montana* has been used for the synthesis of TiO_2 and $CaCO_3$ particles. The SM extract phase controllers were not very active in the case of TiO_2 (both anatase and heterophase brookite/anatase); while they

result more active towards $CaCO_3$ MPs/SM, conferring shape, roughness, defects, edges and reactive functional groups capable of inducing antimicrobial action against the strains investigated in this study. Finally, another future opportunity (especially for the various TiO_2 polymorphs, since they did not show any antimicrobial activity; unlike that highlighted in the presence of $CaCO_3$ MPs/SM) could be the use of microbial- or bacterial-mediated inorganic particle synthesis (see **Scheme S3**) to better control the size, shape, and defectivity of the resulting particles. These future strategies would involve the application of microorganisms, algae, fungi, etc., as biological activators or phase controllers in a biomimetic, template-based synthesis approach [37].

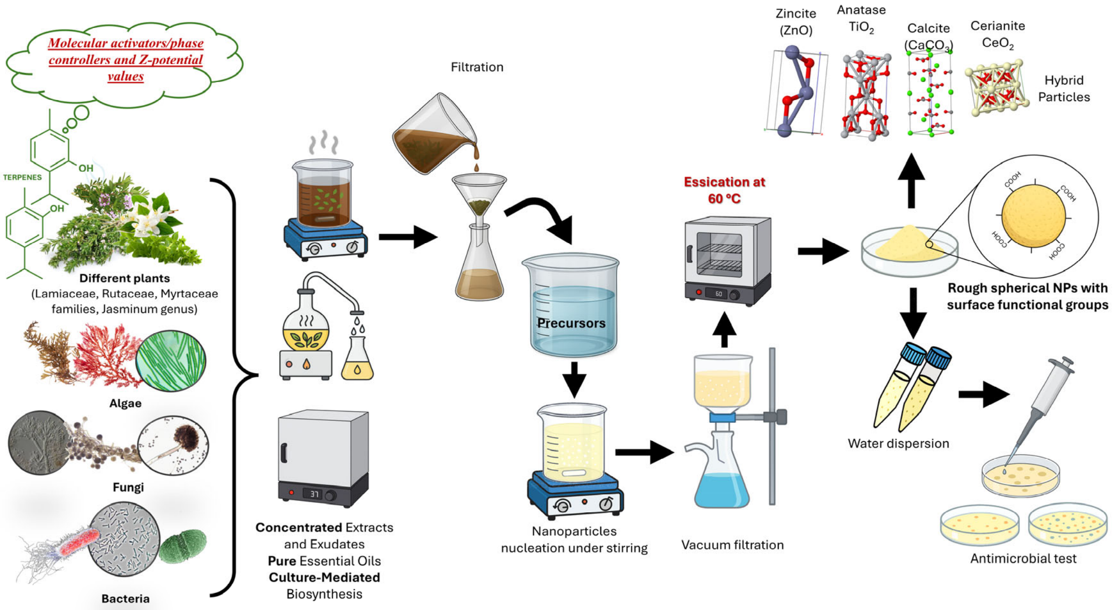

Scheme S3. Optimization synthesis protocol to increase the phase controllers/molecular activators effect/efficiency on several kind of inorganic particles, to improve their antimicrobial and antibacterial performances against selective microorganism strains of interest in numerous clinical-medical, pharmacological, environmental, food, agriculture and Cultural Heritage application fields.

References

1. Kheamrutai Thamaphat; Limsuwan, P.; Boonlaer Ngotawornchai Phase Characterization of TiO_2 Powder by XRD and TEM. *Kasetsart Journal (Natural Science)* 2008, *42*, 357–361.
2. You, Y.F.; Xu, C.H.; Xu, S.S.; Cao, S.; Wang, J.P.; Huang, Y.B.; Shi, S.Q. Structural Characterization and Optical Property of TiO2 Powders Prepared by the Sol-Gel Method. *Ceram Int* 2014, *40*, 8659–8666, doi:10.1016/j.ceramint.2014.01.083.
3. Bellardita, M.; Di Paola, A.; Megna, B.; Palmisano, L. Absolute Crystallinity and Photocatalytic Activity of Brookite TiO2 Samples. *Appl Catal B* 2017, *201*, 150–158, doi:10.1016/j.apcatb.2016.08.012.
4. El-Sheikh, S.M.; Khedr, T.M.; Zhang, G.; Vogiazi, V.; Ismail, A.A.; O'Shea, K.; Dionysiou, D.D. Tailored Synthesis of Anatase–Brookite Heterojunction Photocatalysts for Degradation of Cylindrospermopsin under UV–Vis Light. *Chemical Engineering Journal* 2017, *310*, 428–436, doi:10.1016/j.cej.2016.05.007.
5. Rezaee, M.; Mousavi Khoie, S.M.; Liu, K.H. The Role of Brookite in Mechanical Activation of Anatase-to-Rutile Transformation of Nanocrystalline TiO2: An XRD and Raman Spectroscopy Investigation. *CrystEngComm* 2011, *13*, 5055–5061, doi:10.1039/c1ce05185g.
6. Yan, J.; Wu, G.; Guan, N.; Li, L.; Li, Z.; Cao, X. Understanding the Effect of Surface/Bulk Defects on the Photocatalytic Activity of TiO2: Anatase versus Rutile. *Physical Chemistry Chemical Physics* 2013, *15*, 10978–10988, doi:10.1039/c3cp50927c.

7. Kandiel, T.A.; Robben, L.; Alkaima, A.; Bahnemann, D. Brookite versus Anatase TiO2 Photocatalysts: Phase Transformations and Photocatalytic Activities. *Photochemical & Photobiological Sciences* 2013, *12*, 602–609, doi:10.1039/c2pp25217a.
8. Hu, W.; Li, L.; Li, G.; Tang, C.; Sun, L. High-Quality Brookite TiO2 Flowers: Synthesis, Characterization, and Dielectric Performance. *Cryst Growth Des* 2009, *9*, 3676–3682, doi:10.1021/cg9004032.
9. Anjos, O.; Santos, A.J.A.; Paixão, V.; Estevinho, L.M. Physicochemical Characterization of Lavandula Spp. Honey with FT-Raman Spectroscopy. *Talanta* 2018, *178*, 43–48, doi:10.1016/j.talanta.2017.08.099.
10. Espina, A.; Sanchez-Cortes, S.; Jurašeková, Z. Vibrational Study (Raman, SERS, and IR) of Plant Gallnut Polyphenols Related to the Fabrication of Iron Gall Inks. *Molecules* 2022, *27*, 279, doi:10.3390/molecules27010279.
11. Minteguiaga, M.; Dellacassa, E.; Iramain, M.A.; Catalán, C.A.N.; Brandán, S.A. FT-IR, FT-Raman, UV–Vis, NMR and Structural Studies of Carquejyl Acetate, a Distinctive Component of the Essential Oil from Baccharis Trimera (Less.) DC. (Asteraceae). *J Mol Struct* 2019, *1177*, 499–510, doi:10.1016/j.molstruc.2018.10.010.
12. Vargas Jentzsch, P.; Sandoval Pauker, C.; Zárate Pozo, P.; Sinche Serra, M.; Jácome Camacho, G.; Rueda-Ayala, V.; Garrido, P.; Ramos Guerrero, L.; Ciobotă, V. Raman Spectroscopy in the Detection of Adulterated Essential Oils: The Case of Nonvolatile Adulterants. *Journal of Raman Spectroscopy* 2021, *52*, 1055–1063, doi:10.1002/jrs.6089.
13. Calheiros, R.; Machado, N.F.L.; Fiuza, S.M.; Gaspar, A.; Garrido, J.; Milhazes, N.; Borges, F.; Marques, M.P.M. Antioxidant Phenolic Esters with Potential Anticancer Activity: A Raman Spectroscopy Study. *Journal of Raman Spectroscopy* 2008, *39*, 95–107, doi:10.1002/jrs.1822.
14. Schulz, H.; Özkan, G.; Baranska, M.; Krüger, H.; Özcan, M. Characterisation of Essential Oil Plants from Turkey by IR and Raman Spectroscopy. *Vib Spectrosc* 2005, *39*, 249–256, doi:10.1016/j.vibspec.2005.04.009.
15. Lafhal, S.; Vanloot, P.; Bombarda, I.; Valls, R.; Kister, J.; Dupuy, N. Raman Spectroscopy for Identification and Quantification Analysis of Essential Oil Varieties: A Multivariate Approach Applied to Lavender and Lavandin Essential Oils. *Journal of Raman Spectroscopy* 2015, *46*, 577–585, doi:10.1002/jrs.4697.
16. Rodríguez-Solana, R.; Daferera, D.J.; Mitsi, C.; Trigas, P.; Polissiou, M.; Tarantilis, P.A. Comparative Chemotype Determination of Lamiaceae Plants by Means of GC-MS, FT-IR, and Dispersive-Raman Spectroscopic Techniques and GC-FID Quantification. *Ind Crops Prod* 2014, *62*, 22–33, doi:10.1016/j.indcrop.2014.08.003.
17. Ram Kumar, A.; Selvaraj, S.; Azam, M.; Sheeja Mol, G.; Kanagathara, N.; Alam, M.; Jayaprakash, P. Spectroscopic, Biological, and Topological Insights on Lemonol as a Potential Anticancer Agent. *ACS Omega* 2023, *8*, 31548–31566, doi:10.1021/acsomega.3c04922.
18. Ertani, A.; Pizzeghello, D.; Francioso, O.; Tinti, A.; Nardi, S. Biological Activity of Vegetal Extracts Containing Phenols on Plant Metabolism. *Molecules* 2016, *21*, 205, doi:10.3390/molecules21020205.
19. Yang, M.; Han, L.; Xu, Y.; Ke, H.; Zhou, N.; Dong, H.; Liu, S.; Qiao, G. Near Infrared Spectroscopic Study of Trioctahedral Chlorites and Its Remote Sensing Application. *Open Geosciences* 2019, *11*, 815–828, doi:10.1515/geo-2019-0063.
20. Blessymol, B.; Yasotha, P.; Kalaiselvi, V.; Gopi, S. An Antioxidant Study of Titanium Dioxide (TiO2) Nanoparticles against Mace of Nutmeg in Myristica Fragrans Houtt, Rhizomes of Curcuma Longa Linn and Kaempferia Galanga Extracts. *Results Chem* 2024, *7*, 101291, doi:10.1016/j.rechem.2023.101291.
21. Pushpamalini, T.; Keerthana, M.; Sangavi, R.; Nagaraj, A.; Kamaraj, P. Comparative Analysis of Green Synthesis of TiO2 Nanoparticles Using Four Different Leaf Extract. *Mater Today Proc* 2021, *40*, S180–S184, doi:10.1016/j.matpr.2020.08.438.
22. Ahmadi, O.; Jafarizadeh-Malmiri, H. Intensification Process in Thyme Essential Oil Nanoemulsion Preparation Based on Subcritical Water as Green Solvent and Six Different Emulsifiers. *Green Processing and Synthesis* 2021, *10*, 430–439, doi:10.1515/gps-2021-0040.
23. Agatonovic-Kustrin, S.; Ristivojevic, P.; Gegechkori, V.; Litvinova, T.M.; Morton, D.W. Essential Oil Quality and Purity Evaluation via Ft-Ir Spectroscopy and Pattern Recognition Techniques. *Applied Sciences (Switzerland)* 2020, *10*, 1–12, doi:10.3390/app10207294.
24. Abdallah, R.A.; El-Borady, O.M.; El-Sayed, A.F.; Fawzy, M. A Comparative Study of Chemo- and Phytosynthesized Silver Nanoparticles Using Ceratophyllum Demersum Extract: Characterization and Biological Activities. *Biomass Convers Biorefin* 2025, doi:10.1007/s13399-025-06718-y.
25. Falcioni, R.; Moriwaki, T.; Gibin, M.S.; Vollmann, A.; Pattaro, M.C.; Giacomelli, M.E.; Sato, F.; Nanni, M.R.; Antunes, W.C. Classification and Prediction by Pigment Content in Lettuce (Lactuca Sativa L.) Varieties Using Machine Learning and ATR-FTIR Spectroscopy. *Plants* 2022, *11*, 3413, doi:10.3390/plants11243413.
26. Silverstein, R.M.; Webster, F.X.; Kiemle, D. *Spectrometric Identification of Organic Compounds*; 7th Editio.; Wiley: Hoboken, New Jersey, United States, 2005; ISBN 1118311655.

27. Juhasz-Bortuzzo, J.A.; Myszka, B.; Silva, R.; Boccaccini, A.R. Sonosynthesis of Vaterite-Type Calcium Carbonate. *Cryst Growth Des* 2017, *17*, 2351–2356, doi:10.1021/acs.cgd.6b01493.
28. Zhou, G.T.; Yu, J.C.; Wang, X.C.; Zhang, L.Z. Sonochemical Synthesis of Aragonite-Type Calcium Carbonate with Different Morphologies. *New Journal of Chemistry* 2004, *28*, 1027–1031, doi:10.1039/b315198k.
29. Ramasamy, V.; Anand, P.; Suresh, G. Synthesis and Characterization of Polymer-Mediated CaCO3 Nanoparticles Using Limestone: A Novel Approach. *Advanced Powder Technology* 2018, *29*, 818–834, doi:10.1016/j.apt.2017.12.023.
30. Swain, S.K.; Pradhan, G.C.; Dash, S.; Mohanty, F.; Behera, L. Preparation and Characterization of Bionanocomposites Based on Soluble Starch/Nano CaCO3. *Polym Compos* 2018, *39*, E82–E89, doi:10.1002/pc.24326.
31. Kim, Y.; Caumon, M.C.; Barres, O.; Sall, A.; Cauzid, J. Identification and Composition of Carbonate Minerals of the Calcite Structure by Raman and Infrared Spectroscopies Using Portable Devices. *Spectrochim Acta A Mol Biomol Spectrosc* 2021, *261*, 119980, doi:10.1016/j.saa.2021.119980.
32. Alves, J.F.; Edwards, H.G.M.; Korsakov, A.; de Oliveira, L.F.C. Revisiting the Raman Spectra of Carbonate Minerals. *Minerals* 2023, *13*, 1358, doi:10.3390/min13111358.
33. Legodi, M.A.; De Waal, D.; Potgieter, J.H. Quantitative Determination of CaCO3 in Cement Blends by FT-IR. *Appl Spectrosc* 2001, *55*, 361–365, doi:10.1366/0003702011951786.
34. Vagenas, N. V.; Gatsouli, A.; Kontoyannis, C.G. Quantitative Analysis of Synthetic Calcium Carbonate Polymorphs Using FT-IR Spectroscopy. *Talanta* 2003, *59*, 831–836, doi:10.1016/S0039-9140(02)00638-0.
35. Khalid, A.; Ahmad, P.; Alharthi, A.I.; Muhammad, S.; Khandaker, M.U.; Iqbal Faruque, M.R.; Din, I.U.; Alotaibi, M.A. Unmodified Titanium Dioxide Nanoparticles as a Potential Contrast Agent in Photon Emission Computed Tomography. Crystals 2021, 11, 171. https://doi.org/10.3390/cryst11020171.
36. Zhang, Y.; Wang, H.; Wu, H.; Zhao, X.; Liu, Y. Green Synthesis of Titanium Dioxide Nanoparticles: Characterization, Mechanisms, and Antibacterial Properties of Brookite/Anatase Heterophase Systems. Nanomaterials 2023, 13, 704. https://doi.org/10.3390/nano13040704.
37. Ghareeb, R.Y.; El-Sayyad, G.S.; El-Baz, F.K.; Ahmed, H.S. Recent Trends in Green Synthesis of Metal Nanoparticles Using Natural Resources and Their Biomedical Applications. Microb. Cell Fact. 2024, 23, 341. https://doi.org/10.1186/s12934-024-02609-5.
38. Punz, B.; Christ, C.; Waldl, A.; Li, S.; Liu, Y.; Johnson, L.; Auer, V.; Cardozo, O.; Farias, P. M. A.; Andrade, A. C. D. S.; Stingl, A.; Wang, G.; Li, Y.; Himly, M. Nano-Scaled Advanced Materials for Antimicrobial Applications – Mechanistic Insight, Functional Performance Measures, and Potential towards Sustainability and Circularity. *Environmental Science: Nano* **2025**, *12* (3), 1710–1739. https://doi.org/10.1039/d4en00798k.
